# Interface-related phenomena in epitaxial complex oxide ferroics across different thin film platforms: opportunities and challenges

**DOI:** 10.1039/d2mh01527g

**Published:** 2023-02-21

**Authors:** Judith L. MacManus-Driscoll, Rui Wu, Weiwei Li

**Affiliations:** a Department of Materials Science and Metallurgy, University of Cambridge Cambridge CB3 0FS UK jld35@cam.ac.uk ruiwu001@scut.edu.cn wl337@nuaa.edu.cn; b Spin-X Institute, School of Physics and Optoelectronics, State Key Laboratory of Luminescent Materials and Devices, Guangdong-Hong Kong-Macao Joint Laboratory of Optoelectronic and Magnetic Functional Materials, South China University of Technology Guangzhou 511442 China; c MIIT Key Laboratory of Aerospace Information Materials and Physics, State Key Laboratory of Mechanics and Control of Mechanical Structures, College of Physics, Nanjing University of Aeronautics and Astronautics Nanjing 211106 China

## Abstract

Interfaces in complex oxides give rise to fascinating new physical phenomena arising from the interconnected spin, lattice, charge and orbital degrees of freedom. Most commonly, interfaces are engineered in epitaxial superlattice films. Of growing interest also are epitaxial vertically aligned nanocomposite films where interfaces form by self-assembly. These two thin film forms offer different capabilities for materials tuning and have been explored largely separately from one another. Ferroics (ferroelectric, ferromagnetic, multiferroic) are among the most fascinating phenomena to be manipulated using interface effects. Hence, in this review we compare and contrast the ferroic properties that arise in these two different film forms, highlighting exemplary materials combinations which demonstrate novel, enhanced and/or emergent ferroic functionalities. We discuss the origins of the observed functionalities and propose where knowledge can be translated from one materials form to another, to potentially produce new functionalities. Finally, for the two different film forms we present a perspective on underexplored/emerging research directions.

## Abstract 2

This review focuses on recent progress, current challenges and perspectives in interface-related phenomena of complex oxide ferroic thin films for electronics and energy applications.

## Graphical abstract

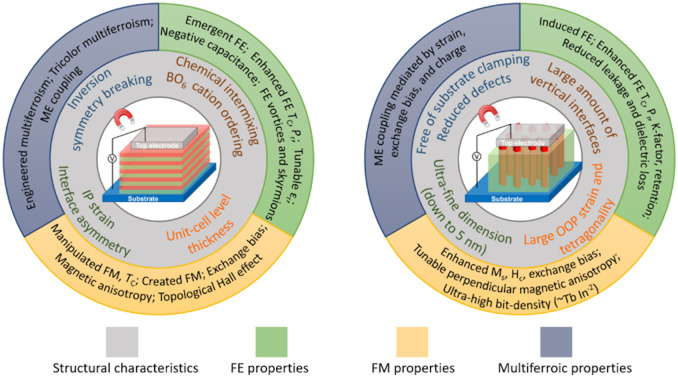

## 1. Introduction

Strongly correlated oxides are fascinating and widely explored functional materials, with an unrivalled range of physical properties. Their most perfect and applicable form is as epitaxial films. To exploit emergent and strain-related interface effects in epitaxial films, typically either superlattice (SL) or vertically aligned nanocomposite (VAN) film forms are studied, each offering different phenomena.

While there have been many important reviews on emergent functional properties of SL^1^ and VAN,^2^ a broader article comparing the phenomena in these different structures has not been presented. The two fields have typically followed quite separate paths by quite separate groups. At first glance, this seems somewhat surprising since the tools for creating the films, the functional materials being studied, and the desired multifunctionalities being aimed for are either the same or are similar. On the other hand, there are subtle differences in approach and knowledge-sets for creating each film form which has precluded overlap between them.

In this review we focus on ferroic properties in the two materials as these are particularly exciting and because they exemplify the salient effects offered by each platform. We aim to present the key differences in the functionalities achieved by exploiting interface effects in the two thin film forms, comparing the merits and drawbacks of each and the reasons for those differences. We also aim to give insight into what can be learned from one form for the benefit of the other. We highlight novel phenomena and promising areas for future studies. We first compare the basic growth and structural features of each form and then we compare their exemplar ferroic properties. After that, we then explore in more detail the individual ferroelectric, ferromagnetic and multiferroic properties of SLs and follow this by exploring the same three phenomena for VANs. After each SL ferroics section, we discuss what can be learned for VAN from the SL field. Where appropriate, we do this also for each VAN section also. Finally, we propose future perspectives for each, including underexplored/emerging research directions.

Ferroics are fascinating exemplar materials to explore for our different film forms as they exhibit very wide ranging and tunable properties with huge future applications prospects. Ferroics refer to materials presenting at least one intrinsic long-range ordering such as magnetization, electric polarization, *etc.* The first application of a ferroic was in lodestone, which was used as a simple magnet and later as a compass, more than 2000 years ago. An understanding of the basic origin of the ferroics was not achieved until the beginning of the last century with the help of quantum mechanics. Over the past century, and in particular in the last decades, many new materials, of metallic, semiconducting, insulating and even organic materials classes, have been added to the ferroics collection. This endows them with very important applications in a range of current electronics devices.

Ferroelectrics have a spontaneous electric polarization that can be switched by an external electric field,^3^ or by mechanical or optical stimuli.^4^ They are used in a wide range of radio-frequency and microwave devices, as well in thermal sensors, actuators, and transducers, *etc.*,^5^ and there is also strong interest for non-volatile memory.^6^ Magnetic thin films are used in magnetic recording media, the leading technology for mass data storage.^7^ Also, magnetic random-access memory (MRAM) could be the winning technology for next-generation ‘universal’ memory.^8^ Another important ferroic form is multiferroics where two ferroic properties coexist, giving promise of magnetoelectricity for multi-state memory in which there is control of magnetism with an electric field. Magnetoelectric random access memory (MeRAM) possesses all advantages of MRAM, but crucially at much lower energy consumption.^9^

For the field of ferroic electronics, thin films of transition metal oxides (TMOs) are of particular interest as they show a very wide range of ferroic properties which are linked to the d orbitals in TM ions. These orbitals, in the presence of a strong ligand field from oxygen ions bonded to the TM ions, produce a wide range of excited states, polarisation effects, and electronic properties. These effects are highly tunable owing to the strong correlation between charge, orbital and structural degrees of freedom of the TM–O bond. Of the TMOs, oxide perovskites have been most widely studied owing to their wide ranging functionalities, ease of growth by pulsed laser deposition (PLD) and compatibility with widely available perovskite single crystals.

If one adds an interface between different TMOs, then symmetry breaking effects, strain and strain-relieving defects^6^ at the interface add yet another handle of tunability, coupling and complexity to TMO functionality. Interfaces and the consequent coupling between two different TMOs can be accomplished effectively in either SL or VAN forms. Conventional SLs have interfaces parallel to the substrate and are grown one layer at a time using vacuum deposition methods. There are a couple of notable exceptions where SLs form by self-assembly from a combined target material, *i.e.* TiO_2_/VO_2_^10^ and YBa_2_Cu_3_O_7−*x*_/BaZrO_3_^11^ the first formed by spinodal decomposition and the second by phase separation.

**Fig. 1:**
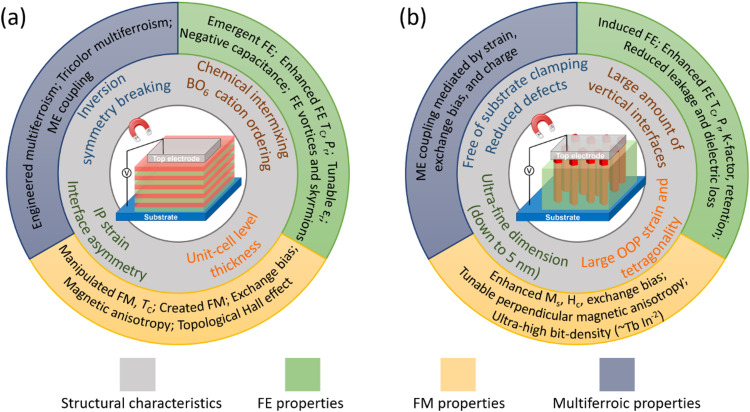
Schematic diagrams showing structural characteristics (inner ring) and observed interface-related phenomena (outer ring) in epitaxial ferroic thin films formed from (a) SL structures and (b) VAN structures. Tricolor multiferroism is a kind of multiferroism induced in a three-component SL. See Fig. 2–8 for more details about how both structural and physical effects link to the observed interface-related phenomena.

The schematics of SL and VAN epitaxial thin film forms, grown mainly by PLD, but less often also by molecular beam epitaxy (MBE) for SL films, are shown schematically in Fig. 1, with structural characteristics and interface-related phenomena shown. For far-from-equilibrium processing methods (including PLD but also sputtering), while vertical columnar grains form in standard planar films, these are different to the VAN columnar grains. For VAN columnar grains, their growth is controlled by vertical epitaxy with the matrix phase in the film and there is vertical epitaxial strain associated with this, as well as potential for interface phenomena at these interfaces, whereas for standard planar films such effects do not occur. The remaining figures in the paper go into more depth than Fig. 1, explaining in detail the origins of the interface phenomena in both VAN and SL films.

We note that multilayer (ML) films are similar to SL films, except they contain fewer layers (<∼5) than SL (>∼5), and the individual film layers are typically thicker than for SL (*e.g.* >15–30 unit cells for ML, and <15 unit cells for SL). In this paper, MLs are discussed where SLs have not yet been presented.

Both SLs and VANs have large area interfaces which enable physical phenomena to be both precisely tuned, *e.g.* using external stimuli such as magnetic or electric fields, and probed, *e.g.* using spectroscopic tools. Table 1 compares the growth and structural features of the two different materials forms.

**Table 1:** Comparison of growth and structural features of SL and VAN epitaxial thin film forms made by PLD or also (although less often) by MBE for SL films

	SL	VAN
Complexity of growth process?	Higher	Lower
Very wide variety of materials combinations possible?	Y	N
Intermixing at interfaces?	Few unit cell level	Unit cell level^g^
Substrate control of strain	Y	N^a^
Easy and uniform control of in-plane strain	Y (in films <20 nm)	N
Easy and uniform control of out-of-plane strain	N	Y (in films >20 nm)^b^
Misfit dislocations at interfaces	N^c^	Y^d^
Dimensionality of interfaces	2D	Non-ideal 2D
Deviation from dimensionality	Very low^e^	Wide^f^

a The substrate influences strain only for ≤20 nm film thickness. Above this thickness, the vertical strain dominates.^12^ Hence, strain can be 3-dimensional with auxetic-like effects possible.^13^

b >1000 nm thicknesses possible.

c Insufficient time in SL to nucleate and grow dislocations between layers so other defects form to alleviate strain.

d Growth of vertical interfaces slow, enabling misfit dislocations to form.^14^

e Interfaces become less 2D-like as the number of superlattice layers increases above ∼10–20 layers and then the layers roughen.

f Interface directions can deviate from the vertical direction, and hence can be tortuous.

g In VAN, there can be chemical segregation of species at an atomic level right at the interface.^15^ This is not the same as chemical intermixing which occurs by reaction between two materials at either side of the interface. This is the case for SL where you have two separate materials grown from separate targets and which can then react at the interface during growth. In VAN, if intermixing is thermodynamically favoured it will have already occurred when the materials are all mixed together in the ceramic target. It means there is not additional chemical reaction/chemical intermixing at the interfaces between the two materials.

The overall drive in research on VANs has been more applied owing to simplicity of growth, and the ability to grow relatively thick, highly strained films resulting from a high density of vertical interfaces, whereas for SL films the focus has been on basic science, enabled by the growth of well-defined 2D interfaces. The growth of SLs is more complex than VAN, requiring reflection high energy electron diffraction (RHEED) and precise substrate heating to carefully control the thickness and qualities of the individual layers. On the other hand, SL growth produces much more well-defined 2D interfaces than VAN, making them ideally suited to exploring emergent interface effects.

VANs grow in a one-shot process from a single ceramic target, and the VAN structure forms epitaxially by self-assembly in the film. The growth of VANs does not need RHEED or very precise control of growth conditions as for epitaxial SLs where each film layer must be very smooth. However, to create new interface-coupled effects in well-aligned VANs, one needs to predict which phases will self-assemble in a given VAN film, starting from the target of all the mixed cations. This requires a greater knowledge of the stabilising forces of epitaxy with the substrate and thermodynamics of the possible phases that can arise from the global cation mix. A simple example is as follows: you may want to grow a VAN of NiO and Fe_3_O_4_ on a standard single crystal substrate. Depending on the Ni : Fe ratio in the target, and assuming standard growth conditions, you will achieve either NiO + NiFe_2_O_4_ or NiFe_2_O_4_ + Fe_3_O_4_, and hence not the desired NiO and Fe_3_O_4_ mix. This example does not mean you will always achieve a ternary phase and a binary phase when the aim is for two binaries. VANs of binaries are indeed possible where intermediate ternaries do not form, *e.g.* TiO_2_ + VO_2_.^16^

The basic ground rules for growing VANs have been set out in comprehensive reviews, the first in 2010^17^ with others building on the understanding subsequently.^18^ Suffice for this article is to note that in VAN films a matrix phase forms in the film with an inclusion phases. The inclusion phase should be structurally dissimilar to the matrix phase to prevent ready mixing of the two materials during growth. This is a different situation to SLs where structurally similar phases are required so that the artificial interfaces that form between them are more perfect. If there is a sufficiently high-volume fraction of the inclusion, it typically forms as nanopillars which grow embedded in the matrix phase. The nanopillars are fine, typically <20 nm in diameter and ∼5–50 nm pitch, controllable by selecting the appropriate growth parameters.^2*b*^

In VAN films the vertical interfaces formed with the matrix can be faceted semi-coherent interfaces or cylindrical surface incoherent interfaces. We term both types of interfaces non-ideal 2D. Owing to the fine pillar dimensions and pitch, it is possible for large vertical and uniform strains to be induced in the matrix from the pillar scaffolds.^17,19,20^ More details about the large vertical strains for VAN films are discussed in Section 6. The most perfect VANs are achieved when the matrix material is structurally the same or very similar to the substrate.^15*b*^ Here, a single interface type (lowest lattice mismatch one) will form, enabling the high density, highly vertical interfaces to be created. For this situation, strain control to >1 micron film thickness has been shown.^14,21^ On the other hand, when the matrix material is lattice mismatched to the substrate and also to the second phase in the VAN film, the films grow less perfectly.^22^ Here, the interfaces have higher energy than when films are isostructural. To minimise the vertical interface energies, a range of different interfaces with similar interfacial energy can form, which leads to meandering of the vertical interfaces. Examples are shown in later figures in this paper. More work needs to be done to give a more quantitative description of VAN microstructures in relation to the properties of the two materials in the film and the growth conditions, and the consequences of the nature of the meandered microstructure on the strain levels in the films and thus the functional properties.

The kinetics of interface formation are very different for SL and VAN films which influences the perfection of the interfaces (and regions in the vicinity of the interfaces). For VAN films the vertical interfaces grow slowly, allowing misfit dislocations to form to partially relieve the strain, whereas for SLs ‘forced’ artificial interfaces form very rapidly, which can lead to the formation of point-like defects to relieve any interfacial strain effects.^14^ Also, since the VAN interfaces are formed at equilibrium and are self-assembled, they are not chemically intermixed as SL film interfaces often are.^1*a*^ This, however, comes at the expense of a lower possible number of materials permutations that can be combined together.

The most enhanced or novel ferroic properties from SL and VAN films, *i.e.* the maximum possible values or novel ferroic properties achieved for each materials form, are summarised in Table 2. Different materials pairs between SL and VAN are compared because different pairs are compatible for each thin film form. Hence for VAN, one typically requires structurally dissimilar materials to prevent a single mixed phase from forming, while for SL structurally similar materials are required to enable high quality interfaces to form. From Table 2, it is clear that the two materials forms offer quite different maximum or novel effects. An important question is what can be learned from one materials platform and translated to another? For example, could a topological Hall effect be achieved in VAN similar to SL, or could a self-biased magnetoelectric effect be achieved in SL similar to VAN?

**Table 2:** Comparison of maximum or novel ferroic properties created using SL and VAN films

	SL	VAN
FE	In 10–200 nm thick films	In ∼micron thick films
	*T*_C_ of BaTiO_3_/SrTiO_3_ increased by several hundred K^23^	*T*_C_ of BaTiO_3_–Sm_2_O_3_ VAN increased by several hundred K^21^
	*P*_r_ ∼ 16.5 μC cm^−2^, the value is doubled in SrTiO_3_/BaTiO_3_/CaTiO_3_*cf.* plain BaTiO_3_ film^24^	*P*_r_ ∼ 12.1 μC cm^−2^, the value is trebled in (Ba,Sr)TiO_3_–Sm_2_O_3_ VAN film ∼ *cf.* plain (Ba,Sr)TiO_3_ film^25^
	Tunability values ∼40% at 200 kV cm^−1^ and 300 K in SrTiO_3_/BaTiO_3_/SrO^26^ which is >3 higher than for BaTiO_3_ plain films	Tunability values are ∼75% at 200 kV cm^−1^ and 300 K in (Ba,Sr)TiO_3_–Sm_2_O_3_ VAN film which is ∼40% higher than for (Ba,Sr)TiO_3_ plain film.^27^ Also, loss scales with tunability, which is opposite to plain films.
	Negative capacitance observed in Ba_0.8_Sr_0.2_TiO_3_/LaAlO_3_ and PbTiO_3_/SrTiO_3_ at 300 K^28^	Negative capacitance not yet observed
	Polar vortices observed in PbTiO_3_/SrTiO_3_^28*b*,29^	Polar vortices not yet observed

FM	In-plane exchange bias as high as 3 kOe induced in CaRuO_3_/CaMnO_3_ at 10 K^30^	Perpendicular exchange bias of ∼0.91 kOe at room temperature obtained in NiO–NiFe_2_O_4_ VAN^22^*cf.* ∼0.1 kOe in NiO–NiFe_2_O_4_ bilayer at room temperature.^31^
	High interfacial magnetization of up to 2.5*μ*_B_ (396 emu cm^−3^) achieved in LaFeO_3_/LaCrO_3_^32^	Saturation magnetisation in Fe_3_O_4_–BiFeO_3_ VAN film ∼900 emu cm^−3^*cf.* ∼300 emu cm^−3^ in pure Fe_3_O_4_ film with similiar thickness^33^
	High *T*_C_ ferromagnetism of up to 375 K created at interfaces from non-magnetic parent materials in LaFeO_3_/LaCrO_3_^32^	Ferro/ferrimagnets with several-hundred-*K T*_C_ created from AFM parent material ZnFe_2_O_4_^34^
	Perpendicular magnetic anisotropy (*K*_eff_) of 4 × 10^6^ erg cm^−3^ (∼13 kOe) realized in La_1−*x*_Sr_*x*_MnO_3_/SrIrO_3_ at 10 K^35^	Perpendicular magnetic anisotropy of 85 kOe induced in BaTiO_3_–CoFe_2_O_4_ at 300 K *via* strain^36^
	Topological Hall effect up to 75 μΩ cm observed in LaMnO_3_/SrIrO_3_ at 10 K^37^	No topological Hall effect observed
	DME coefficient *α*_ME_ up to 55 V cm^−1^ Oe^−1^ at 300 K observed in BaTiO_3_/BiFeO_3_^38^	DME coefficient *α*_ME_ up to 20 V cm^−1^ Oe^−1^ at 300 K measured in BiFeO_3_–CoFe_2_O_4_^39^

ME	No sizable CME yet observed	Self-biased CME effect at room temperature with ME coefficient of ∼1.4 × 10^−9^ s m^−1^ at 300 K achieved in Na_0.5_Bi_0.5_TiO_3_–NiO–NiFe_2_O_4_^40^

The rest of the paper expands on the information in Table 2, discussing the different ferroic phenomena of ferroelectricity, ferromagnetism and multiferroicity observed in SL films (part A) and VAN films (part B), with potential synergies between the two film forms noted. Finally, an overall summary, perspective and conclusions are presented (part C).

## A. Ferroics in superlattice films

## 2. Ferroelectric phenomena in superlattice films

Ferroelectric polarization in oxide perovskites of the ABO_3_ formula is induced by polar displacement in B-site cations at the centres of BO_6_ octahedral cages, or displacement of A-site (lone-pair active) cations which sit between the octahedra. In SL films, a wide range of fascinating ferroelectric properties have been observed, *e.g.* ferroelectricity induced at the interfaces between two non-ferroelectric systems, enhanced *T*_C_s and higher remanent polarization values, improved dielectric tunability, negative capacitance effects, and polar skyrmions/vortices. In the following, we provide details of exemplar recent systems where each property has been observed and, in some cases, also manipulated, and we explain how the strain, interfaces, and defects in the SLs contribute to these properties. In Section 6, we compare the same effects in VAN films.

### 2.1 Induced ferroelectricity in superlattice films

**Fig. 2:**
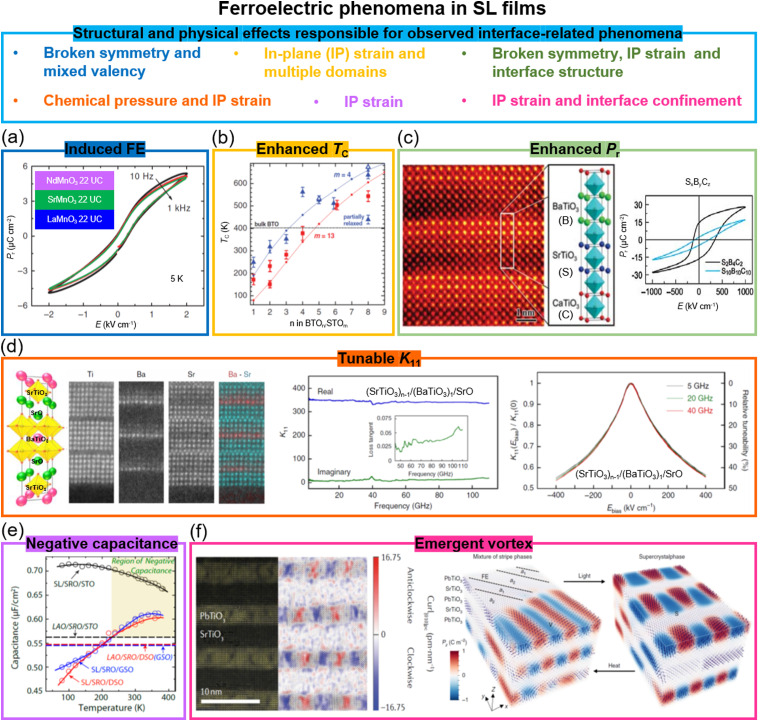
Ferroelectric phenomena in SL films. (a) *P*–*E* hysteresis loops measured from (NdMnO_3_)_*n*_/(SrMnO_3_)_*n*_/(LaMnO_3_)_*n*_ superlattices. Reproduced with permission.^41^ Copyright 2012 Springer Nature. (b) FE *T*_C_ in (BaTiO_3_)_*n*_/(SrTiO_3_)_*m*_ superlattices as a function of *n* and *m* layer numbers. Reproduced with permission.^23^ Copyright 2006 AAAS. (c) Left panel: STEM image and diagram of (SrTiO_3_)_*x*_/(BaTiO_3_)_*y*_/(CaTiO_3_)_*z*_ superlattices. Right panel: *P*–*E* hysteresis loops measured from (SrTiO_3_)_2_/(BaTiO_3_)_4_/(CaTiO_3_)_2_ and (SrTiO_3_)_10_/(BaTiO_3_)_10_/(CaTiO_3_)_10_ superlattices. Reproduced with permission.^24^ 2005 Springer Nature. (d) Left: Schematic of crystal structure of (SrTiO_3_)_*n*−1_/(BaTiO_3_)_1_/SrO superlattices and STEM images the Ti, Ba, Sr and (Ba + Sr) elements. Middle: Real and imaginary parts of dielectric constant (*K*, the ratio of the material's permittivity *ε* to the permittivity of vacuum *ε*_0_) as a function of frequency for the *n* = 6 superlattice film. The inset shows the film loss tangent. Right: The ratio of *K*(*E*_bias_, applied electrical field)/*K*(0, without applied electrical field) (left-hand axis) and relative tunability (right-hand axis) of the *n* = 6 superlattice film. Reproduced with permission.^26^ 2020 Springer Nature. (e) Temperature dependent capacitance for superlattices grown on SrTiO_3_, DyScO_3_, and GdScO_3_ substrates. Reproduced with permission.^28*a*^ Copyright 2014 American Chemical Society. (f) Left panel: STEM image of vortex structures formed in a (SrTiO_3_)_10_/(PbTiO_3_)_10_ superlattice. Reproduced with permission.^29*a*^ 2016 Springer Nature. Right panel: Optical and thermal field control of the coexistence phase and superlattice phase. Reproduced with permission.^42^ 2019 Springer Nature.

Remarkably, in several systems SLs formed of two different non-ferroelectric perovskite layers have given rise to emergent ferroelectricity.^43^ For example, in SLs of non-ferroelectric (NdMnO_3_)_*n*_/(SrMnO_3_)_*n*_/(LaMnO_3_)_*n*_, ferroelectricity was created below 40 K (Fig. 2a), tunable by varying SL periodicity.^41^ The emergent ferroelectricity was ascribed to broken space inversion symmetry and the Mn^3+^/Mn^4+^ mixed valency.

No such emergent interface FE has been reported in VAN films to date. While it is certainly harder to grow such perfect vertical interfaces of desired compositions in VAN films, it is possible in some systems where the matrix film is highly similar in both structure and lattice parameter to the substrate.^44^ In fact, the effect has likely never been searched for, and is a fruitful avenue of future research, particularly in manganite-based VAN systems where mixed Mn valence is induced by the presence of oxygen vacancies. Indeed, interface structures in VAN systems predicted using DFT calculations show strongly distorted oxygen sublattices and higher oxygen vacancy concentrations.^15*b*^ Hence, in a SrMnO_3_/CeO_2_ VAN film, interface-induced oxygen vacancies are anticipated. These would reduce some of the Mn^4+^ to Mn^3+^. Hence, it is possible that emergent FE could arise from this mixed valency in VAN films, just as in SL films. The signal would, in theory, be much higher than in SL films, owing to the higher density of interfaces. We note that a reduced overall cation valence at interface structures in VAN films would not be limited to manganites. Other transition metal oxides would also likely give mixed valent cation interfaces and hence wide-ranging novel interfacial properties. This is a rich area for future exploration.

Another proven route to inducing ferroelectricity in SLs is to induce octahedral rotations into a SL layer from another SL layer. This leads to hybrid improper ferroelectricity.^45^ It has been shown in perovskite SLs with the same *B* ions but different *A* (*A* and *A*′) ions, hence *A*/*A*′ SLs, *e.g.* LaGaO_3_/YGaO_3_. The octahedral rotations induce a sizeable polarization of 0.65 μC cm^−2^. This same effect would likely not be possible in VAN films, owing to the larger lateral pitch of the vertical structures, *i.e.* in the range 5–50 nm, rather than <1–3 nm as required here. We note also that ferroelectricity across the interfaces in the SLs is predicted to originate from charge transfer,^46^ charge order,^47^ and element intermixing.^48^ Similar effects are likely also taking place in VAN structures but, as far as known, have not been probed yet. This would be an interesting area of future research.

### 2.2 Enhanced ferroelectric *T*_C_ and remanent polarization, *P*_r_, in superlattice films

(BaTiO_3_)_*n*_/(SrTiO_3_)_*m*_ SL with *n* = 1 (single unit cell thick BaTiO_3_) and *m* = 30 grown on (001) SrTiO_3_, with 2.3% lattice mismatch in the BaTiO_3_,^23,49^ gives a ferroelectric *T*_C_ of 250 K,^23^*i.e.* lower than the bulk value of 403 K (Fig. 2b), which means the SrTiO_3_ is effective in straining the BaTiO_3_ and the atomically thick nature of the BaTiO_3_ does not seriously degrade the ferroelectricity. Furthermore, by tuning *n* and *m*, the *T*_C_ of the BaTiO_3_ is increased up to 638 K. The higher-than-bulk *T*_C_ arises from the strain effect applied to the BaTiO_3_ layers by the SrTiO_3_ (Fig. 2b).^23^ A similar approach, using thin ferroelectric and non-ferroelectric perovskite layers, has been applied to other FE SL combinations,^50^ both bilayers and even trilayers. A trilayer example is shown in Fig. 2c. Here *x* unit cells of SrTiO_3_, *y* unit cells of BaTiO_3_, and *z* unit cells of CaTiO_3_ (S_*x*_B_*y*_C_*z*_) were grown on (001) SrTiO_3_ substrates.^24^ The strain in BaTiO_3_ layers is fully retained when its thickness does not exceed the combined thickness of the other layers. As a consequence, an overall 50% enhancement of the SL global remanent polarization, *P*_r_, is achieved (Fig. 2c). Apart from the strain effect, the broken inversion symmetry and specific interface structure play unexpected roles in the enhancement of ferroelectric polarization. The broken inversion symmetry in three-component SLs has been used for investigating the ferroelectric ground states, the dielectric properties,^51^ and multiferroicity, as we discuss more in Section 4.

As we show later, the *T*_C_ in VAN films is highly tunable, and, just as for SL films, several hundred K *T*_C_ enhancements are readily achievable. The biggest differences with SLs is the ease of growth of VAN films combined with the ability to maintain vertical strain to > micron thicknesses,^21,25^ whereas in SLs the total film thicknesses are only several 10's unit cells maximum. VANs are therefore more suited to actuator/energy harvesting/dielectric applications where larger volumes of material are needed, whereas SL films are more suited to Si-based electronics where charges at planar interfaces play an important role in switching for processor or memory applications.

### 2.3 Dielectric property enhancements in superlattice films

Owing to temperature/frequency-stable permittivity and dielectric tunability, dielectric materials are of interest in applications where device miniaturization and high-energy-density storage are needed.^52^ (Ba,Sr)TiO_3_ is an established compound that has been used in the development of tuneable dielectrics in microwave filters. There is no ferroelectric transition for pure SrTiO_3_ as it is paraelectric in bulk, but the ferroelectric *T*_C_ is 403 K for BaTiO_3_. The *T*_C_ is in between these values by varying the Ba : Sr ratio. However, charged pointed defects in (Ba, Sr)TiO_3_ thin films, notably oxygen vacancies, lead to increased dielectric loss.^53^

(SrTiO_3_)_*n*−1_(BaTiO_3_)_1_SrO SLs with *n* = 6 (single unit cell thick BaTiO_3_) (Fig. 2d) have lower dielectric loss than standard films. This is because the single unit cell thick BaTiO_3_ in the SL is under chemical pressure from the SrTiO_3_ layers. This likely reduces oxygen loss at the same time as increasing the tetragonality and hence also the ferroelectric polarization. Overall, at a temperature *T* = 300 K and an electric field *E* = 400 kV cm^−1^, a dielectric constant (*K*) tunability, *i.e.* the ratio of *K*(*E*)/*K*(0) (right panel in Fig. 2d), of around 46%, and a loss tangent (tan *δ*) of ∼0.06 were achieved at a 110 GHz frequency.^26^ Higher tunability values were achieved in much thicker (∼μm) VAN films (Section 6), *i.e.* dielectric constant tunability of up to 75% and tan *δ* of 0.01,^27,54^ although the frequency studied so far was lower than for SL films, at ∼1 MHz.

Another form of ferroelectricity in SLs has been demonstrated *via* improper ferroelectricity. In (PbTiO_3_)_*n*_(SrTiO_3_)_*m*_ SLs with very short periods of *n* = 2 and *m* = 3, improper ferroelectricity^55^ is achieved resulting from strong competition between ferroelectric PbTiO_3_^39^ and antiferrodistortive SrTiO_3_^56^ distortions across the interface, causing antiferrodistortive rotations of the oxygen atoms in PbTiO_3_. In turn, a relatively high dielectric constant of 600 is achieved at 300 K and, from 300 K to 550 K, the dielectric constant is independent of temperature. No improper ferroelectricity has been observed in VAN films to date but there is the possibility to observe it in the most perfect VAN structures. The PbTiO_3_/SrTiO_3_ VAN system would be a good starting point. While phase intermixing between PbTiO_3_ and SrTiO_3_ could occur in the VAN films, there is precedent for lack of mixing between similarly lattice matched perovskite VAN materials.^57^ Thus for PbTiO_3_/SrTiO_3_ VAN, a similar situation could come about. Less (or no) mixing compared to the bulk thermodynamic case related of the role of epitaxy at the substrate film interface. Hence pure SrTiO_3_ on SrTiO_3_ is the lowest interfacial energy situation and may prevent intermixing with Pb. More experiments and calculations are highly required to confirm this possibility.

### 2.4 Negative capacitance and emergent polar vortex in superlattice films

The negative capacitance effect in ferroelectrics occurs when there is a negative differential capacitance around a zero charge level. Hence, an increase in charge in the ferroelectric occurs when the voltage is decreased. This effect could reduce the subthreshold swing in a conventional transistor below the otherwise minimum limit of 60 mV decade^−1^.^28*a*,58^ Negative capacitance in SL films of ferroelectric Ba_0.8_Sr_0.2_TiO_3_/dielectric LaAlO_3_^28*a*^ has been shown with the capacitance enhancement level tuned by epitaxial strain *via* the growth of SLs on three different substrates (Fig. 2e). Furthermore, in PbTiO_3_/SrTiO_3_ SLs a direct measurement of steady-state negative capacitance has shown that local regions of negative capacitance emerge at domain walls, where the energy density is larger than that in the bulk of the domains and the polarization is suppressed.^58*a*^

Another novel phenomenon observed recently in PbTiO_3_/SrTiO_3_ SLs is the formation of polar vortices in PbTiO_3_. In fact, ferroelectric vortex states were theoretically predicted in nanostructures,^59^ but not observed in thin films until 2016. Long-range and chiral ferroelectric vortex structures have been observed in PbTiO_3_ in (PbTiO_3_)_*n*_/(SrTiO_3_)_*n*_ SLs with *n* = 10 (left in Fig. 2f).^29*a*^ A combination of substrate induced strain and confinement from the SrTiO_3_ layers produces the effect leading to a clockwise vortex-like pattern, paired with every other vortex in an anti-clockwise manner. The coexistence phase of ferroelectric *a*_1_/*a*_2_ domains and vortex states can be transferred to a single 3D SL phase by optical and thermal manipulation (right in Fig. 2f).^42^ Room-temperature polar-skyrmion bubbles have further been observed in these SL systems,^28*b*^ giving the possibility of highly efficient skyrmion memory.

There have been no such reports thus far of polar vortices in VAN films. However, the ∼5 nm length scales, strain and confinement effects involved in the above SL example, can also be readily achieved in VAN films, even in the PbTiO_3_/SrTiO_3_ system. Hence, PbTiO_3_ pillars should form in a SrTiO_3_ matrix when the film is grown on SrTiO_3_. The Pb and Sr would be unlikely to mix based on the differing ion sizes and charges, and also because it would not be preferred because pure SrTiO_3_ forms a perfect epitaxial match on SrTiO_3_. Thus, it may be possible to achieve few nm sized nanopillars of PbTiO_3_ as can be achieved in other VAN systems^60^ and thus create vertical polar skyrmions throughout the whole film thickness.

## 3. Magnetic phenomena in superlattice films

Similar to ferroelectric oxide thin films, magnetic oxides, notably transition metal oxide (TMO) perovskites, have potential applications for data storage and non-volatile random-access memory. Strong magnetization, high *T*_C_, and perpendicular anisotropy are highly desired for such applications. A broad range of functionalities arising from the interplay between the interconnected degrees of freedom of the magnetic spins, lattice structure, bonding orbitals and ionic charges, mediated by corner-shared BO_6_ units octahedra which can tilt around three orthogonal axes^61^ can be exploited and manipulated in SLs.

**Fig. 3:**
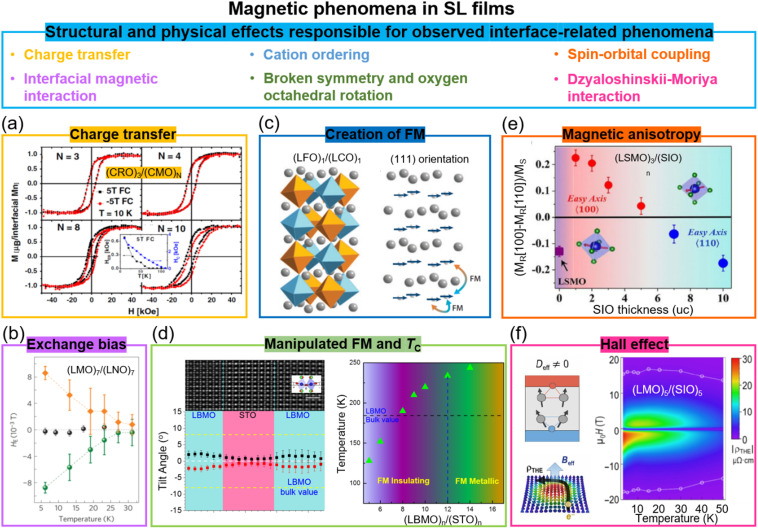
Magnetic phenomena in superlattice films. (a) Magnetic hysteresis loops of (CaRuO_3_)_3_/(CaMnO_3_)_*N*_ superlattices. Reproduced with permission.^30^ Copyright 2012 American Physical Society. The inset shows the temperature dependent *H*_C_ and *H*_EB_ for (CaRuO_3_)_3_/(CaMnO_3_)_8_. (b) Temperature dependent *H*_E_ for (LaNiO_3_)_7_/(LaMnO_3_)_7_ superlattices after field-cooling in a +0.4 T field (green circles) and in a −0.4 T field (orange diamonds). Reproduced with permission.^62^ Copyright 2012 Springer Nature. (c) Schematic diagram of (LaFeO_3_)_1_/(LaCrO_3_)_1_ superlattices grown on (111)-oriented substrates and the resultant spin structures. Reproduced with permission.^63^ Copyright 2014 Annual Reviews. (d) Left panel: STEM image (top) and octahedral tilt angles (bottom) of (La_0.9_Ba_0.1_MnO_3_)_8_/(SrTiO_3_)_8_ superlattice. Right panel: Ferromagnetic *T*_C_ as a function of layer thickness in (La_0.9_Ba_0.1_MnO_3_)_*n*_/(SrTiO_3_)_*n*_ superlattices. Reproduced with permission.^64^ Copyright 2020 Wiley-VCH. (e) Magnetic anisotropy of (La_0.7_Sr_0.3_MnO_3_)_3_/(SrIrO_3_)_*n*_ superlattices as a function of SrIrO_3_ layer thickness. Reproduced with permission.^65^ Copyright 2016 PNAS. (f) Left panel: Schematic diagram of the topological Hall effect originating from the Berry phase and a chiral spin texture. Right panel: The topological Hall resistivity of the (LaMnO_3_)_5_/(SrIrO_3_)_5_ superlattice. Reproduced with permission.^37^ Copyright 2020 AAAS.

At SL interfaces, the following effects have been shown: the enhancement of *T*_C_, modified electronic conduction, induced ferromagnetism from non-ferromagnetic layers, exchange bias, magnetic anisotropy, and topological Hall effect, *etc.* (Fig. 3). In Section 7, we discuss the comparative magnetic effects in VAN films.

### 3.1 Magnetic interactions across interfaces involving 3d TMO superlattice films

Exchange bias (EB) has been induced in SLs *via* two different effects. First, charge transfer (to equilibrate the differing chemical potentials of carriers in the materials on either side of the interface) occurs in many oxide SLs and leads to new electronic and magnetic states.^66^ For example, in (CaRuO_3_)_3_/(CaMnO_3_)_*N*_ SLs composed of the antiferromagnetic insulator CaMnO_3_ and the paramagnetic metal CaRuO_3_, an exchange bias as high as 3 kOe is induced at 10 K (Fig. 3a).^30,67^ Here, a ferromagnetic interface is created in one unit cell, because of the double exchange interaction in the CaMnO_3_ at the interface where Mn^4+^ is partially reduced to Mn^3+^ because of electron transfer from CaRuO_3_ to CaMnO_3_. The consequent ferromagnetic/antiferromagnetic coupling across the interface creates the exchange bias effect.

Exchange bias is also achieved in (111)-oriented LaMnO_3_/LaNiO_3_ (ferromagnetic/paramagnetic) SLs (Fig. 3b). Here, interfacial interactions, *e.g.* quantum confinement effects,^68^ induce a complex magnetic structure in a non-magnetic material. Hence, magnetism resembling a spin density wave in the paramagnetic LaNiO_3_ layers in contact with the ferromagnetic LaMnO_3_ is created.^62^ An exchange bias as high as 90 Oe is induced at 5 K. Large vertical exchange bias effects have been demonstrated at much higher temperatures in VAN films, namely at room temperature, and the reasons for this are discussed in Section 7.

Similar to the (CaRuO_3_)_3_/(CaMnO_3_)_*n*_ SLs, in (LaMnO_3_)_2*n*_/(SrMnO_3_)_*n*_ SL, a metallic ferromagnetic state emerges at the interface from the parent materials which are not ferromagnetic, namely an antiferromagnetic Mott insulator (LaMnO_3_),^69^ and a band insulator with G-type antiferromagnetism (SrMnO_3_).^70^ Again, interfacial charge transfer between Mn^3+^ and Mn^4+^ cations is responsible for emergent FM, *i.e.* electrons ‘leak’ from the Mn^3+^ in LaMnO_3_ into the Mn^4+^ in SrMnO_3_.^70,71^ The metallic state is apparent for *n* ≤ 2 (when the occupied Mn e_g_ electrons spread over the entire lattice), but for higher *n*, the bulk of the film (insulating antiferromagnetic) dominates the properties.

Emergent ferromagnetism and metallicity should also be easily achievable in VAN films, as charge transfer across interfaces is not restricted to planar interfaces. To date, in several VAN systems interesting additional magnetic and conducting effects have been observed (separate to the expected contributions from the bulk properties to the film).^15*a*,72^ However, the origins of these effects, whether they be from charge transfer effects, orbital reconstructions, *etc.*, have not been widely explored. Further studies of such effects in perfectly aligned VAN structures, including understanding of the phenomena using high resolution microscopic and scanning probe tools to probe the structures and interface charge states is a fruitful area of future research. Also, since the vertical interfaces in VAN films intersect the film surface, the conducting properties of VAN interfaces are readily measurable giving an advantage over SL where interfaces are buried.^73^

Another mechanism to induce ferromagnetism at interfaces is by tuning the relative fractions of B–O–B, B–O–B′, and B′–O–B′ bonds, In (ABO_3_)/(AB′O_3_) SLs, there are different magnetic exchange interactions couplings across the interfaces for different orientation films. Hence, different spin structures have been observed for (LaFeO_3_)_1_/(LaCrO_3_)_1_ SLs grown on (001)-, (110)-, and (111)-oriented SrTiO_3_ substrates.^32*a*,74^ As shown in Fig. 3c, based on Anderson, Goodenough, and Kanamori rules, ferromagnetic exchange interactions across Fe–O–Cr bonds can be realized in the (111)-oriented SL films. A high *T*_C_ of 375 K and high interfacial magnetization of 2.5*μ*_B_ were achieved in (111)-oriented (LaFeO_3_)_1_/(LaCrO_3_)_1_ SL.

A further interfacial phenomenon which induces modified interfacial properties in magnetic perovskite SLs is orbital reconstruction. This occurs when TMO thin films are strained leading to different BO_6_ octahedral rotations.^61,64^ For example, the ferromagnetic *T*_C_ in La_2/3_Sr_1/3_MnO_3_ is significantly enhanced from 370 K in bulk La_2/3_Sr_1/3_MnO_3_ to 650 K in (La_2/3_Sr_1/3_MnO_3_)_3_/(BaTiO_3_)_3_ SLs owing to straining of the La_2/3_Sr_1/3_MnO_3_ by BaTiO_3_.^75^ The change of the Mn orbital occupation from d_*x*^2^−*y*^2^_ within the film to d_3*z*^2^−*r*^2^_ at the interface is responsible.

A further mechanism for tuning the magnetic coupling within SL layers containing magnetic moments is *via* non-trivial oxygen octahedral coupling (OOC) across the heterointerfaces which produces different BO_6_ octahedral rotations in the layer of interest.^61*c*,76^ For instance, in non-magnetic (LaMnO_3_)_*n*_/(SrTiO_3_)_*n*_ SLs, magnetisation was induced in the interfacial layers in LaMnO_3_ with a maximal saturation magnetization of 2.97*μ*_B_ per Mn for the *n* = 6 SL film.^77^ It should be noted that while interactions between layers in the films lead to the modulation of BO_6_ octahedral rotations, epitaxial strain also play an important role. To largely eliminate the strain effect, (La_0.9_Ba_0.1_MnO_3_)_*n*_/(SrTiO_3_)_*n*_ SLs were studied,^64^ where there is only 0.2% strain between the La_0.9_Ba_0.1_MnO_3_ and SrTiO_3_. It was possible to engineer the BO_6_ octahedral rotations up to 12 unit cells, larger than previous reports by 6 unit cells. Consequently, the out-of-plane Mn d_3*z*^2^−*r*^2^_ orbitals became occupied, the Mn e_g_ bandwidth reduced, a ferromagnetic insulating state in La_0.9_Ba_0.1_MnO_3_ created, and *T*_C_ enhanced to 235 K from 185 K in bulk (Fig. 3d).

As already noted, in SL films the substrate plays a role in controlling the perovskite octahedral rotations and oxygen octahedral coupling. The substrate exerts strain on the film which induces octahedral rotations in the film, but there is also coupling of the film octahedra to substrate octahedra. The question is whether similar interface coupling effects could be created in VAN films but without the interference from the substrate as is the case of SL films. In fact, in VAN the substrate influence is weak above around 20 nm film thickness and since micron thick films are relatively easy to grow and indeed preferred for VAN, any coupling effects across the vertical interfaces in VAN are largely independent of the substrate. On the other hand, as shown in Table 1, the challenge for VAN is creating highly faceted interfaces which are also perpendicular to the substrate without meandering. As already noted, this is highly possible when the matrix material is the same as the same or a very closely lattice and structurally matched material as the substrate material, *e.g.* SrTiO_3_-based VAN film on SrTiO_3_ substrate,^54^ and hence there is wide scope for exploring new interface coupling effects in VAN films with the most ideal interfaces.

### 3.2 Magnetic interactions across interfaces involving in 5d TMO superlattice films

SLs containing perovskites with strong spin–orbit coupling have been investigated to further explore new interface coupled effects and exotic magnetic reconstructions. For example, in SrMnO_3_/SrIrO_3_ superlattices,^78^ where SrMnO_3_ is the 3d antiferromagnetic insulator and SrIrO_3_ is a 5d paramagnetic metal, there is charge transfer of ∼0.5 electrons per unit cell from SrIrO_3_ to SrMnO_3_. Hence, there is induced ferromagnetism in the SrMnO_3_ which is normally antiferromagnetic.^79^ In addition, the strong spin–orbit coupling also leads to perpendicular magnetic anisotropy (PMA) and a large anomalous Hall effect. Furthermore, in La_0.7_Sr_0.3_MnO_3_/SrIrO_3_ (ferromagnetic metal/paramagnetic metal) SLs,^65^ magnetic coupling across the interfaces produces an emergent weak ferromagnetism in the SrIrO_3_ layers. Also, since magnetocrystalline anisotropy is closely linked to spin–orbit coupling, the in-plane magnetic anisotropy of La_0.7_Sr_0.3_MnO_3_ was changed by varying the thickness of the SrIrO_3_ (Fig. 3e). A PMA constant (*K*_eff_) of 4 × 10^6^ erg cm^−3^ was further optimised by increasing the doping level, *x*, in the La_1−*x*_Sr_*x*_MnO_3_/SrIrO_3_ SLs.^35^

Broken inversion symmetry and strong spin–orbit coupling in 3d–5d interfaces can also lead to large chiral Dzyaloshinskii–Moriya interactions (DMIs), inducing non-trivial spin textures, *i.e.* topological phenomena. In LaMnO_3_/SrIrO_3_ SLs (Fig. 3f), a highly robust chiral magnetic phase with large topological Hall effect (THE) of 75 μΩ cm was observed.^37^ The present research on the interfaces with iridate oxide systems is still in infancy, and many open and intriguing questions still remain to be explored.

Finally, we note that the skyrmion structures formed in SrIrO_3_ superlattices incorporate insulating or metallic layers are formed in films of only 2–8 unit cells (or 1–4 nm) thickness.^37,80^ Thus, as mentioned for the polar skyrmions in Section 2.4, it is highly possible to grow VAN structures of this dimension and potentially grow, by self-assembly, skyrmion SrIrO_3_ columns in a film matrix, the route for growing perovskite nanocolumns in VAN films having already been demonstrated in several systems, *e.g.* BiFeO_3_, BaTiO_3_, and Ba_1−*x*_Sr_*x*_TiO_3_ systems.^25^ Indeed, it is possible that SrIrO_3_ would grow more readily in VAN films compared to plain epitaxial films. This is because the phase could be stabilised using vertical epitaxy with a matrix phase in the VAN film.^19*b*^ Also, to maintain stoichiometry of the SrIrO_3_, a matrix material containing either or both Sr and Ir species could be used so as to produce a source material to prevent loss of these volatile cations during growth.

## 4. Multiferroism and magnetoelectricity in superlattice films

Materials that exhibit simultaneous order in their electric and magnetic ground states are promising for enabling electric field control of magnetism *via* the CME effect. Such materials are rare, however, due to the contradicting requirements of empty d orbitals *via* cation off-centering required for ferroelectricity and partially filled d orbitals for magnetism.^81^ Creating composite systems where the properties of two different systems are coupled across their interface is a potential way to create artificial multiferroics. Oxide SLs are one form of composite system. As we discuss below, when two systems are coupled *via* an interface in SLs comprising magnetic and ferroelectric layers, proximity effects, broken inversion symmetry, strain and defects, and geometric frustration and strain engineering can give rise to either multiferroism (at least two ferroic properties in *one* materials systems) and/or artificial magnetoelectricity (electric field control of magnetism or *vice versa*). We note that while there have been many predictions of the aforementioned effects in a range of SLs^82^ not many have been experimentally verified. On the other hand, there have been interesting experimental observations in SLs not predicted by theory, typically using combinations of a perovskite which exhibits ferroelectricity, with another which exhibits some form of magnetic order.

**Fig. 4:**
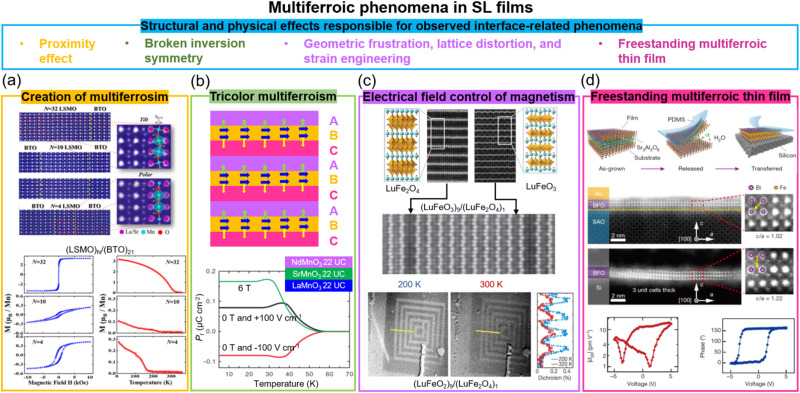
Multiferroic phenomena in SL films. (a) Top panel: STEM images of (La_2/3_Sr_1/3_MnO_3_)_*N*_/(BaTiO_3_)_21_ superlattices. Bottom panel: *M vs. H* loops (left) and *M vs. T* curves (right) for (La_2/3_Sr_1/3_MnO_3_)_*N*_/(BaTiO_3_)_21_ superlattices. Reproduced with permission.^83^ Copyright 2017 PNAS. (b) Top panel: A tricolor superlattice for designing the multiferroicity. Reproduced with permission.^84^ Copyright 2007 Elsevier. Bottom panel: Temperature dependent polarization measured under electrical and magnetic field for NdMnO_3_/SrMnO_3_/LaMnO_3_ superlattices. Reproduced with permission.^41^ Copyright 2012 Springer Nature. (c) Top panel: STEM images of end-members LuFe_2_O_4_ (left) and LuFeO_3_ (right); middle panel: (LuFeO_3_)_9_/(LuFe_2_O_4_)_1_ superlattice. Bottom panel: XMCD PEEM images of (LuFeO_3_)_9_/(LuFe_2_O_4_)_1_ superlattice measured at 200 K and 320 K. Reproduced with permission.^85^ Copyright 2016 Springer Nature. (d) Top panel: Schematic of growth and transfer of a freestanding thin films. Middle panel: STEM images of 3-unit-cell BiFeO_3_ film before and after etching Sr_3_Al_2_O_6_ layer. Bottom panel: Piezoelectric properties of 4-unit-cell BiFeO_3_ film after etching Sr_3_Al_2_O_6_ layer. Reproduced with permission.^86^ Copyright 2019 Springer Nature.

Two key examples of multiferroism induced in SLs are in La_2/3_Sr_1/3_MnO_3_/BaTiO_3_ (non-polar-tilt ferromagnet/polar ferroelectric) and La_2/3_Sr_1/3_MnO_3_/BiFeO_3_ (non-polar-tilt ferromagnet/ferroelectric antiferromagnet). In the first case (Fig. 4a), due to proximity effects a structural phase transition from non-polar to unconventional polar (hence giving both ferroelectricity and ferromagnetism, thus multiferroicity) was induced in ultrathin 4-unit-cell La_2/3_Sr_1/3_MnO_3_.^83^ In the second case, magnetism (up to 200 K) was induced in ultrathin 5-unit-cell ferroelectric BiFeO_3_,^87^ to a level of 1.83 ± 0.16*μ*_B_ per Fe, increased from 0.03*μ*_B_ per Fe in bulk BiFeO_3_, and attributed to strong orbital reconstruction between Fe and Mn across the interfaces.

The creation of ferroelectricity, multiferroicity, and a DME effect has been achieved through simultaneous breaking of time-reversal and spatial-inversion symmetries in “tricolour” ABCABC SLs composed of three sequential kinds of perovskite layers^88^ (top plot in Fig. 4b). The polarization at the AB (or BC) interfaces has the same sign leading a non-vanishing toroidal moment equivalent to a built-in vector potential under the presence of spin–orbit coupling, thereby producing ME coupling. For example, a ferroelectric *T*_C_ of 40 K was achieved in NdMnO_3_/SrMnO_3_/LaMnO_3_ SLs, even though individual layers have no polar moment. Also, ME coupling was achieved by magnetic modulation of the polarization (bottom plot in Fig. 4b).^41^ Density functional calculations show that the combination of broken space inversion symmetry and the presence of Mn^3+^/Mn^4+^ mixed valence, arising from cationic asymmetry and interfacial polar discontinuity, produce emergent ferroelectric and ME coupling. However, the interface-induced ME coupling is very weak and occurs below 55 K.

Other works on creating magnetoelectricity in SLs have centred around a wide range of SL combinations, *e.g.* in BaTiO_3_/BiFeO_3_ (ferroelectric/ferroelectric and antiferromagnet) SLs a direct ME coefficient *α*_ME_ up to 55 V cm^−1^ Oe^−1^ at 300 K was measured,^38*b*^ higher than in single-phase BiFeO_3_ (*α*_ME_ = 4.2 V cm^−1^ Oe^−1^), which was explained by the diffusion of Ba and Ti into the BiFeO_3_ layers. Hence, the influence of unwanted chemical intermixing across the interface and impurities buried within superlattices is uncertain.

Another different mechanism to achieve multiferroicity and ME coupling was demonstrated using symmetry breaking across a SL interface of (LuFeO_3_)_*m*_/(LuFe_2_O_4_)_1_. Rumpling (periodic buckling of lattice planes) in LuFeO_3_^89^ imposed a local distortion in one unit cell LuFe_2_O_4_, thus removing the mirror symmetry in the LuFe_2_O_4_ and enabling ferroelectricity to emerge in this ferrimagnetic phase^85^ (Fig. 4c). The ferrimagnetic *T*_C_ of the LuFe_2_O_4_ was increased from 240 K to 281 K because of a local distortion applied by LuFeO_3_. Since the ferroelectric order couples to the ferrimagnetism, electrical field control of magnetism, as measured using X-ray magnetic circular dichroic photoemission electron microscopy (XCMD PEEM), was achieved at 200 K (Fig. 4c). The clamping of the films to the substrate or leakage effects possibly limit macroscopic measurements using electrodes applied across the whole film.

Finally, we note that there has been more success in achieving direct ME (DME) in all-metallic systems^90^ than in oxides. An example is (Ta/Cu/IrMn/FeCoSiB)_*n*_/AlN/Si,^90*c*^ where a relatively large direct ME effect of 96 V cm^−1^ Oe^−1^ was observed at 300 K. However, the requirement to use precious metal Ir, the need to use a relatively complex stack of layers, and no observation of CME, are limiting factors for this system. Hence, arguably, there is more promise for oxide systems, although controlled interface composition and structure, and the elimination of substrate clamping effects are important areas of future study. The latter effect prevents intrinsic displacement of ferroelectric and magnetostrictive materials.

Very promisingly, in recent years much progress has been made with lift-off and transfer of films to practical substrates.^86,91^ As far as known, the transfer process has not been applied to multiferroic SL films but it has been used for single layer films and it is expected that multiferroic SL films would behave similarly. The advantage of lifting off a multiferroic SL film is that it would be free from substrate clamping. This could enable strain coupled magnetoelectricity to be realised. Fig. 4d shows an example of using water-soluble Sr_3_Al_2_O_6_ as the sacrificial buffer layer to produce freestanding BiFeO_3_ multiferroic thin films with a high crystalline quality that can be fabricated down to one unit cell. Also, robust and switchable ferroelectric polarization was demonstrated for a four unit-cell, transferred BiFeO_3_ film on Si, as confirmed by STEM and piezoelectric measurements, respectively. We note that VAN films have also been successfully lifted-off and transferred to Si. Owing the nanoscale nature of VAN films, they appear to be more resistant to crack propagation yielding a higher success rate of film transfer compared to plain films.^92^ More work is needed to explore the performance of lifted-off ferroic and multiferroic VAN films.

Contrasting with much of the SL work describe above, artificial multiferroic effects in VAN films have not focused on purely interfacial phenomena, but on more macroscopic effects caused by strain coupling of phenomena across vertical interfaces which take effect in the whole body of film. An advantage of VAN films over SL is the lack of a substrate clamping effect. On the other hand, leakage currents can be problematic if the right combinations of materials are not used. Strategies for overcoming the leakage while also boosting interface coupling are discussed for VAN multiferroics in Section 8.

## 5. Summary of ferroic phenomena in superlattice films

Overall, artificial SLs of complex oxides for ferroics are systems which provide an excellent platform for fundamental research, allowing the validity of theoretical predictions to be probed, new science to be revealed, and the realization of new materials with tailor-made properties. On the other hand, SLs also have challenges as interfaces can be subject to intermixing effects and defect formation which are hard to probe and identify. Promisingly, improvements in tools to understand interfaces are improving rapidly, there are also continuous advances in growth tools. Hence, SL films continue to have huge promise for next generation electronics.

## B. Vertically aligned nanocomposite (VAN) films

## 6. Ferroelectric phenomena in VAN films

In SL films (discussed in part A above), it has been demonstrated that ferroelectricity can be induced from interfacing non-FE materials, and also that fascinating effects such as negative capacitance and polar vortices can be created. As already noted, such phenomena have not yet been observed in VAN films (Table 2). On the other hand, a number of other structural effects (Table 1) give rise to fascinating phenomena in VAN films. In particular, novel strain effects play a strong role in determining functional properties.

We recall that in standard planar SL ferroelectric films, epitaxial strain from the underlying substrate is often used to tune properties.^1*e*,24,93^ However, this substrate-induced interfacial strain can only extend to tens of nanometers thickness and hence the *T*_C_ enhancement is largely confined to the interfacial region. It has been shown in epitaxial perovskites that from above the first tens of nanometers thickness up to ∼100 nm, the strain is relieved by a range of crystalline defects to compensate the strain.^94^ Such defects can degrade the functionality of epitaxial perovskite devices which exploit the functionality of the whole film rather than just the interface, *e.g.* a strain-enhanced-*T*_C_ ferroelectric actuator. In VAN films, on the other hand, by incorporating stiff nanopillars into a ferroelectric matrix large vertical strains (of >1%) can be induced throughout the whole film and hence the thickness-limiting substrate strain effect is not relied upon.^21^ This enables wide-ranging ferroelectric property tuning in micron thick films. Since the lateral distance between the nanopillars is typically only ∼20 nm, the strain is not relaxed to a large extent as for epitaxial films, and so the film perfection and crystallinity remains high for 100s nm thick VAN films.^13,95^ Hence, enhanced *T*_C_, remnant polarization, retention, dielectric tunability, and reduced loss tangent and leakage current are achieved.^25,27,54,96^

In section A, we presented several examples of VAN compositions that could be explored to try and mimic and also new opportunities the exciting emergent effects achieved in SL films. In the following sections we focus on VAN films. Before, we go into details of each of the enhanced properties, explaining how the strain in VAN films contributes to these properties, we first consider how research on SLs could benefit from VAN research. A key processing advantage of the VAN approach over SL is it is relatively fast and simple for exploring new combinations of materials and their interfaces. Hence, VAN can serve as fast screening method to predict which phases and which ratios of phases could also give rise to novel interface related functionalities in SL structures. For example, in Section 8, we show how a triple VAN system enables a self-biased magnetoelectric to be achieved. For SL films, it would take longer to explore the right thicknesses of the layers to use, to be sure there are no interface mixing effects, and also to know if the magnetoelectric effect were present (as it could obscured by substrate clamping effects). Hence, with the knowledge gained from VAN, SL films of the demonstrated compositions could now be tested, and films lifted off to remove any clamping effects.

### 6.1 Enhancement of ferroelectric *T*_C_, remanent polarization, *P*_r_, piezoelectric *d*_33_ and retention in VAN films

**Fig. 5:**
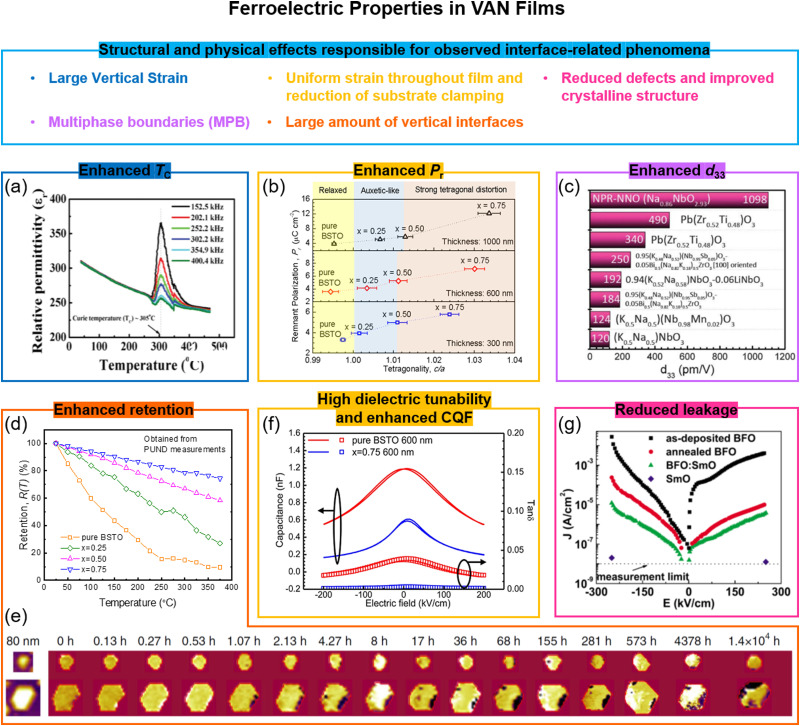
Enhanced ferroelectric properties achieved using VAN structures. (a) Cross-sectional TEM image of SrTiO_3_–Sm_2_O_3_ VAN; (b) remnant polarization of (Ba,Sr)TiO_3_–Sm_2_O_3_ VAN film as a function of the tetragonality of (Ba,Sr)TiO_3_ phase. Reproduced with permission.^25^ Copyright 2017 Wiley-VCH. (c) Giant piezoelectric Na-deficient NaNbO_3_–NaNbO_3_ (NPR-NNO) VAN film. Reproduced with permission.^97^ Copyright 2020 AAAS. (d) Comparison of normalized retained polarization *versus* temperature for a pure (Ba,Sr)TiO_3_ film and for (Ba,Sr)TiO_3_–Sm_2_O_3_ VAN films with different Sm_2_O_3_ contents (*x*). Reproduced with permission.^25^ Copyright 2017 Wiley-VCH. (e) The evolution of polarisation retention in a FE domain of a BiFeO_3_ (111) mesocrystal with time. Reproduced with permission.^96*b*^ Copyright 2016 Springer Nature. (f) High tunable relative permittivity, *ε*_r_, as shown by the large change in capacitance value with electric field in a (Ba,Sr)TiO_3_–Sm_2_O_3_ VAN film, measured at 1 MHz. An enhanced commutation quality factor (CQF) is also achieved. Reproduced with permission.^27^ Copyright 2012 American Chemical Society. (g) Reduced leakage current of a BiFeO_3_–Sm_2_O_3_ VAN film compared to plain BiFeO_3_ or plain Sm_2_O_3_ films. Reproduced with permission.^96*a*^ Copyright 2008 AIP Publishing.

The first demonstration of enhanced ferroelectricity using VAN was in ∼2 micrometre-thick BaTiO_3_-based VAN films embedded with stiff Sm_2_O_3_ pillars yielding a >2% vertical extension in the BaTiO_3_. While many BaTiO_3_ applications are limited by its relatively low *T*_C_ (∼396 K),^98^ here in the VAN films a *T*_C_ up to ∼970 K (higher also than the 638 K achieved in BaTiO_3_/SrTiO_3_ SL^99^) can be achieved which opens up many more applications possibilities.^21^ Other BaTiO_3_-based and SrTiO_3_-based VAN films have also shown strong enhancements with *T*_C_ enhanced (Fig. 5a) and leakage current reduced.^54,96*c*,100^ We note that reduced leakage is not universal for VAN films but depends on whether the interface is in tension or compression,^72*b*^ the nature of the interface structure(s) that form in the VAN film to minimise interfacial energy,^73*b*^ the nature of the transition metal cations and their redox equilibria, and whether a rectifying p–n junction forms between the film and substrate.^40^

As shown in Table 2, similar ferroelectric property enhancements are only achievable in planar or SL films if they are ∼10's nm thick or less.^93*a*^ Such high *T*_C_s in micron thick films are necessary for advanced device applications, such as the electrocaloric devices,^101^ actuators,^102^ and microwave tuneable filters,^53*a*^ and cannot be achieved in planar films.

The remnant polarization (*P*_r_) has also been systematically increased using (Ba,Sr)TiO_3_-based VAN structures (Fig. 5b), both by increasing the fraction, *x*, of nanopillars and increasing film thickness (to ∼μm's),^25^ both of which increase the film tetragonality. The *P*_r_ values are about ∼3× larger compared to (Ba,Sr)TiO_3_ planar films (Table 1). A near doubling of *P*_r_ has also been shown in BaTiO_3_–NiO VAN films.^103^ A giant enhancement of piezoelectric response is also possible in VAN films (Fig. 5c). Liu *et al.*^97^ showed in nanopillar-like sodium-deficient NaNbO_3_ VAN a piezoelectric coefficient *d*_33_ of ∼1098 pm V^−1^, which compares to ∼22 pm V^−1^ for stoichiometric NaNbO_3_ films. The *T*_C_ of the VAN film is ∼723 K, which is similar to that of the stoichiometric NaNbO_3_ film. Local heterogeneity of sodium-deficient NaNbO_3_ and stoichiometric NaNbO_3_ phases was deemed to be responsible.

Temperature-dependent and time-dependent changes of the polarization state of ferroelectric films, *i.e.* thermal and temporal retention, are of great importance for non-volatile nanoelectronic devices based on ferroelectrics,^25,96*b*,104^*e.g.* for above-room temperature-operation, high-stability memory devices. The retention parameters are sensitive to film thickness and microstructure, electrodes, *etc.*^105^ Much enhanced thermal and temporal polarization retention has been demonstrated in (Ba,Sr)TiO_3_–Sm_2_O_3_ and CoFe_2_O_4_–BiFeO_3_ VAN films (Fig. 5d and e) compared to the pure film counterparts of (Ba,Sr)TiO_3_ and BiFeO_3_, respectively. Ultra-dense vertical interfaces lead to a strong tetragonal distortion and vertical ferroelectric domains^25,96*b*^ which are ‘pinned’, thus retarding relaxation processes.

### 6.2 Dielectric property enhancements and reduced leakage in VAN films

Stemming from the increased tetragonality induced in the ferroelectric phase, dielectric properties are enhanced in VAN films.^27^ In (Ba,Sr)TiO_3_-based VAN films, a tunability of 75% (200 kV cm^−1^ field), and a low dielectric loss of 0.01 was achieved at room temperature, in contrast to the respective values of ∼52% and 0.06 in pure (Ba,Sr)TiO_3_ films^27^ (Fig. 5f). Critically, the loss is reduced when the tunability is enhanced which is opposite to planar films and results from the unusual strain states in VAN films.^13^ The combination of high tunability and low loss lead to a high communication quality factor (CQF) value. In SrTiO_3_-based VAN films, a record communication quality factor (CQF) of up to 3300 was achieved which gives the system strong potential for tuneable RF applications.^54^

Leakage is a long-standing problem in ferroelectric oxide thin films, commonly caused by oxygen deficiency and the presence of point or line defects. As already mentioned above, leakage can be reduced using VAN films. Taking the example of BiFeO_3_, a promising candidate for multifunctional non-volatile memory devices because of the coexistence of both the antiferromagnetic order with Néel temperature (*T*_N_) of 643 K^106^ and ferroelectric order with *T*_C_ of 1123 K,^107^ leakage from a range of defects is common and can give either p-type or n-type behaviour.^108^ In BiFeO_3_-based VAN films, the leakage was reduced by several orders (Fig. 5g).^72*a*,96*a*^ This was ascribed to the compressive out-of-plane (OOP) strain of −1.46% that was induced in the BiFeO_3_, induced by the embedded nanopillars. It was postulated that such a compression would reduce the oxygen vacancy concentration and hence carriers associated with these vacancies.^109^ However, some chemical intermixing, leading to doping of Sm into the BiFeO_3_ could also contribute to the lowering of the leakage. Of course, not all VAN films can have judicious compression engineered into them as for this BiFeO_3_ example.

### 6.3 Exploiting both bulk and surface effects for property enhancements using VAN

One key advantage of VAN films over SL or standard films is the ability to create very high surface area structures from them, and at the same time to retain strong ferroic properties even after treating them by chemical etching. Such etching removes the pillar phase in the VAN rendering a mesoporous film. There are many potential applications which could benefit from mesoporous films with tubular pores from the etched nanopillars, having high surface area for chemical, gas, or biological sensing, or even filtration (especially because the pore size is tunable *via* growth parameters and VAN film composition). Adding in ferroicity to the mesoporous film could enable greater sensitivity or tunability of the device performance/operation.

**Fig. 6:**
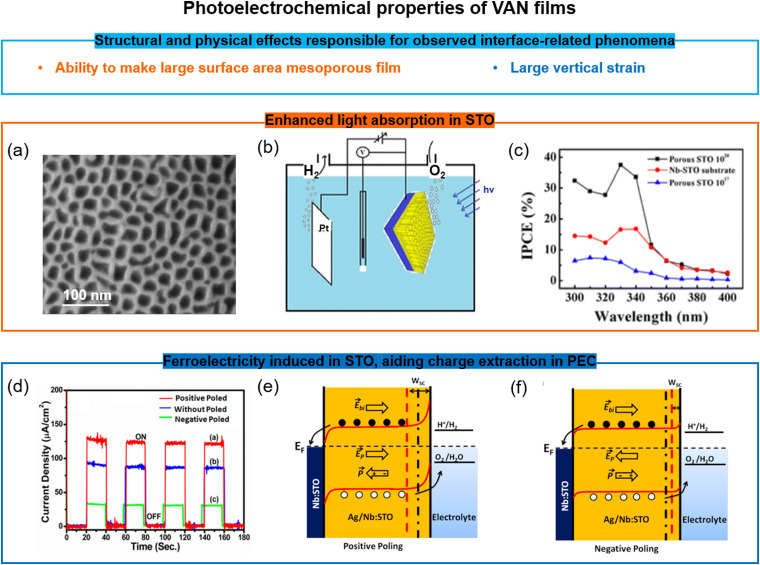
Doped/undoped SrTiO_3_-based VAN porous ferroelectric films for water splitting applications. (a) SEM image of the surface of a undoped mesoporous SrTiO_3_ film. Reproduced with permission.^110^ Copyright 2016 American Chemical Society. (b) Schematic of the water-splitting by the undoped mesoporous SrTiO_3_ film. Reproduced with permission.^110^ Copyright 2016 American Chemical Society. (c) Enhanced incident photon to current efficiency (IPCE) in the undoped mesoporous SrTiO_3_ film compared to both a continuous SrTiO_3_ film and the Nb-doped SrTiO_3_ substrate. Reproduced with permission.^110^ Copyright 2016 American Chemical Society. (d) The photoelectrochemical (PEC) response of a mesoporous (Ag,Nb)-co-doped SrTiO_3_ film at different polarization states. Reproduced with permission.^111^ Copyright 2019 American Chemical Society. (e and f) Schematic of the modified band alignment at the interfaces in the mesoporous (Ag, Nb)-co-doped SrTiO_3_ film and electrolyte solution with different polarization states and the impact on the PEC. Reproduced with permission.^111^ Copyright 2019 American Chemical Society.

One example of the benefits of mesoporous VAN is the doped/undoped SrTiO_3_–MgO VAN film system. Here, the MgO is chemically etched out of the film, leaving a mesoporous SrTiO_3_ matrix (Fig. 6a) with the out-of-plane tensile strain in the SrTiO_3_ matrix largely intact. More understanding of the mechanisms and universality of matrix strain retention after etching pillars out of the VAN films is required. This mesoporous matrix leads to photoelectrochemical (PEC) water splitting compared to literature (Fig. 6b and c),^110^ arising from (a) an order of magnitude larger surface area for light absorption, (b) optimised carrier concentration from Ag and Nb of the doped SrTiO_3_,^111^ and (c) above-room-temperature ferroelectricity from the vertical strain effect induced by the VAN structure, which produced a reduced flat-band potential and enhanced charge extraction (Fig. 6d–f)^110,111^

Another area which has been unexplored so far is the filling of epitaxial mesoporous films with different phases, *e.g.* using atomic layer deposition or solution processing. This can produce new composites with metallic or polymeric phases which can couple to the mesoporous matrix either to achieve multifunctional properties, or to simply exploit large surface areas and reduced dimensionality (important for strong light absorption and high charge extraction in optoelectronic systems, for example). These areas remain largely unexplored but there is strong potential for new multifunctional systems to emerge.

## 7. Magnetic phenomena in VAN films

While novel magnetic phenomena involving a range of interactions have been widely explored at interfaces in SL films, direct interactions at interfaces in VAN films has been limited to exchange bias coupling.^112^ However, a wide range of other interactions may be present at VAN interfaces, just as at SL interfaces, *e.g.* charge transfer, orbital reconstructions, and magnetic exchange interactions. However, so far, they remain unexplored. On the one hand, while non-planar interfaces in VAN might frustrate some of these interactions, on the other VAN films have perfectly clean interfaces and this is beneficial in terms of not degrading the interactions.

A key benefit of VAN over SL resides in the potential for high density magnetic memory and storage applications. Radically new approaches are required to increase magnetic recording densities beyond 1 Tbit in^−2^ and to lower energy consumption.^113^ While self-assembled magnetic nanoparticles, such as the self-assembled FePt nanoparticle arrays shows a high area density up to 50 Tbit in^−2^ for <4 nm particle sizes,^114^ difficulties exist for anchoring and ordering these nanoparticles. Self-assembled VAN films, with ∼Tbit in^−2^ density (∼10 nm sized features with ∼10 nm spacing) represent a promising approach. The vertical alignment of magnetic nanocolumns anchored in a non-magnetic matrix gives exchange decoupled, well-separated perpendicular magnetic recording bits. A range of other enhanced magnetic properties in VAN films also have potential benefit for magnetic storage/memory applications, as outlined below.

### 7.1 Enhanced magnetization in VAN films

Magnetization can be enhanced significantly in VAN films. In BiFeO_3_ (90%)–Fe_3_O_4_ (10%) nanocomposite films, enhanced saturation magnetization values of 900 emu cm^−3^ were obtained compared to 300 emu cm^−3^ for pure Fe_3_O_4_ films. The result was explained by a highly strained (OOP, 7%), ultra-thin Fe_3_O_4_ phase in which ferromagnetism rather than ferrimagnetism was favoured.^33^

Similar magnetisation enhancement was found in Pr_0.5_Ba_0.5_MnO_3_–CeO_2_ VAN films^115^ due to the enhanced tetragonality in Pr_0.5_Ba_0.5_MnO_3_ from the stiff CeO_2_ nanopillars, although other structural effects could be at play. However, in La_0.7_Sr_0.3_MnO_3_–MgO VAN films, the magnetization was suppressed, possibly because of octahedral tilting/rotations.^116^

**Fig. 7:**
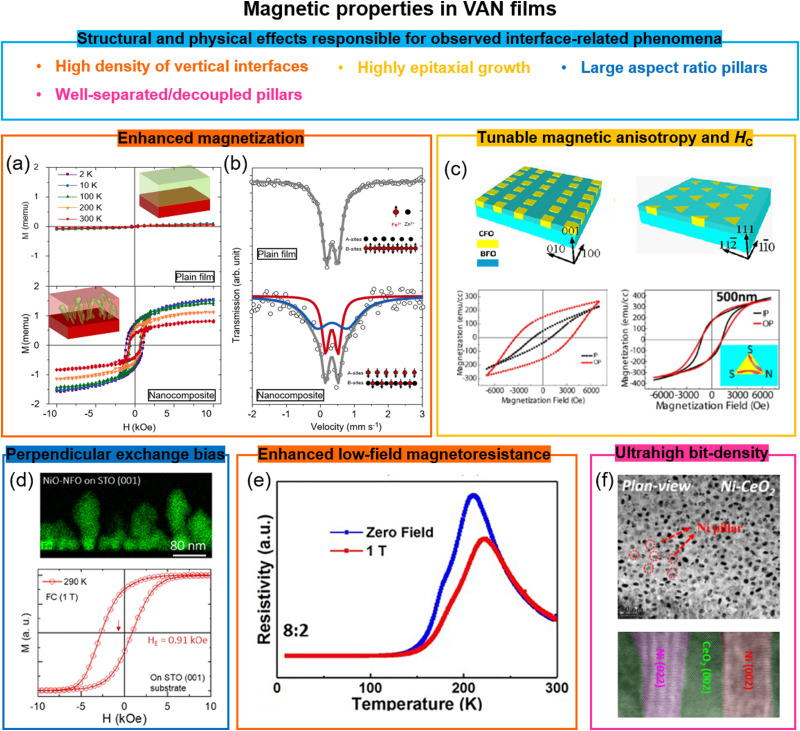
Enhanced magnetic properties achieved using VAN structures. (a) The magnetic hysteresis loops of a ZnFe_2_O_4_ plain film and a ZnFe_2_O_4_–SrTiO_3_ VAN film grown on a SrTiO_3_ (001) substrate, measured at different temperatures. The magnetic field was applied in the OOP direction. Reproduced with permission.^34^ Copyright 2018 Wiley-VCH. The insets show schematics of a plain ZnFe_2_O_4_ film and a ZnFe_2_O_4_–SrTiO_3_ VAN film, respectively. (b) Mössbauer spectra of the Fe element in the ZnFe_2_O_4_ plain film and the ZnFe_2_O_4_–SrTiO_3_ VAN film. The insets show schematics of the magnetic structures of the ZnFe_2_O_4_ phases. Reproduced with permission.^34^ Copyright 2018 Wiley-VCH. (c) Tunable magnetic anisotropy and coercivity in BiFeO_3_–CoFe_2_O_4_ VAN films grown on SrTiO_3_ substrates with magnetic field applied either in-plane (IP) or OOP. Reproduced with permission.^120^ Copyright 2013 American Chemical Society. (d) Enhanced perpendicular exchange bias effect in a NiO–NiFe_2_O_4_ VAN film with magnetic field applied OOP. Reproduced with permission.^22^ Copyright 2018 American Chemical Society. (e) Temperature dependence of the resistivity measured at different magnetic fields applied OOP. Reproduced with permission.^121^ Copyright 2019 AIP Publishing. (f) Plan-view (top panel) and cross-sectional (bottom panel) high resolution TEM images of a CeO_2_–Ni VAN film. Reproduced with permission.^122^ Copyright 2016 Royal Society of Chemistry.

Antiferromagnetic-to-ferromagnetic property switching has also been demonstrated in different VAN systems, *e.g.* in spinel ZnFe_2_O_4_ based VAN films,^34^ a *T*_C_ of ∼500 K was achieved with 4.8*μ*_B_ per Fe moment, and moderate coercivity (Fig. 7a (bottom panel)), overwhelmingly larger than the *T*_N_ (∼10 K) of bulk ZnFe_2_O_4_ (Fig. 7a) (top panel). The VAN films induced stronger intersite A–B exchange interactions of the Fe^3+^ cations than the intrasite interactions at the A- or B-sites. Mössbauer spectra (Fig. 7b) showed that half of the Fe^3+^ ions go into the A sites in the ZnFe_2_O_4_ phase. This is similar to the case for nanocrystalline powder form of ZnFe_2_O_4_.^117^

Similar antiferromagnetic-to-ferromagnetic property switching has also been also observed in other VAN films, *e.g.* SmMnO_3_–Bi_2_O_3_,^96*d*^ (Sr,Sm)MnO_3_–Sm_2_O_3_,^118^ and EuTiO_3_–Eu_2_O_3_.^119^ In all cases, large vertical strains were induced, *e.g.* in the latter case the value was ∼3.15% in the EuTiO_3_ phase. The strain decreases the Eu–Ti–Eu bond angle along 〈111〉 up to 1°, leading to a weakening of the antiferromagnetic interactions and switching from antiferromagnetic to ferromagnetic behaviour.

### 7.2 Highly tunable magnetic anisotropy and coercivity in VAN films

The magnetic anisotropy defines the direction of the magnetization in magnetic materials. In continuous magnetic thin films, the anisotropy is usually dominated by shape anisotropy, which makes the in-plane (IP) direction the easy plane for the magnetization to minimize the demagnetization energy. However, perpendicular magnetic anisotropy (PMA) is required to achieve ultra-high-density data recording.^123^ In traditional media, PMA is obtained in the ultrathin films and films with noble metals or rare-earth elements with strong spin–orbit coupling, such as Co/Pt multilayers^124^ and DyCo_5_,^125^*etc.* In magnetic oxide films, PMA is typically generated using epitaxial strain-induced magnetostrictive anisotropy^126^ or it is driven by symmetry mismatch^127^ at the interfaces which leads to distinct orbital reconstructions. However, due to the interfacial nature of PMA, in standard planar films (including SL), the effect degrades with film thickness above a few nm to tens of nm depending on the mechanism for the PMA.

In VAN films the total anisotropy is controlled by three kinds of magnetic anisotropy, *i.e.* magnetocrystalline, shape and magnetostrictive. While magnetocrystalline anisotropy is an inherent property of the material, shape and magnetostrictive anisotropy are highly tunable in VAN structures. Because of the large aspect ratio of the magnetic nanopillars which grow in VAN films, a large shape anisotropy is produced with an easy axis along the OOP direction, producing the PMA. A shape anisotropy field *H*_shape_ = 2.0 kOe was estimated in the BaTiO_3_–CoFe_2_O_4_ VAN films, where the aspect ratio of magnetic pillars was around 10.^36^ The shape anisotropy is controlled by substrate orientation (as well as other growth parameters). The substrate orientation effect on the magnetic anisotropy and coercivity has been demonstrated for CoFe_2_O_4_ pillars in BiFeO_3_–CoFe_2_O_4_ VAN films (Fig. 7c) as well as also for other VAN systems.^120,128^ Since BiFeO_3_ is an antiferromagnet, it has been proposed that spin-flop exchange coupling between the compensated BiFeO_3_ and the CoFe_2_O_4_ spins at the interfaces might contribute to the observed PMA in BiFeO_3_–CoFe_2_O_4_ VAN films.^129^

Strain-induced stress anisotropy in VAN films also plays a significant role *via* the magnetoelastic effect. Here, the magnetoelastic energy depends on both the magnetostriction coefficient of the magnetic material as well the strain in it. CoFe_2_O_4_ has a large magnetostrictive coefficient with a large strain-induced magnetoelastic effect. In BaTiO_3_–CoFe_2_O_4_ VAN films, a compressive strain in the CoFe_2_O_4_ columns of ∼0.8% is achieved, with corresponding anisotropy field of *H*_stress_ = 34 kOe, matching the coercivity of the measured magnetic hysteresis.^130^ The magnetostrictive and magnetocrystalline anisotropies have been systematically tuned by varying the chemical composition of the Co_*x*_Ni_1−*x*_Fe_2_O_4_ column phases in the Co_*x*_Ni_1−*x*_Fe_2_O_4_–BiFeO_3_ VAN system.^131^ Large tunability of magnetic anisotropy and coercivity has also been achieved in La_0.7_Sr_0.3_MnO_3_–MgO VAN, where a switch from the easy-plane magnetic anisotropy to PMA occurs as the volume ratio of MgO increases, thus leading to different strain values in the La_0.7_Sr_0.3_MnO_3_ matrix.^60*a*^

Potential systems for high-density perpendicular recording media have also been shown in ferromagnetic metal pillar-oxide VAN systems.^132^ Favourable properties such as ultra-high bit densities of ∼Tb in^−2^ (Fig. 7f shows an example for a CeO_2_–Ni film),^133^ uniaxial anisotropy, strong OOP magnetization, high coercivities, anisotropic electric conductivity,^132*b*^ and higher *T*_C_s and lower intrinsic resistances than ferromagnetic oxides^133,134^ have all been demonstrated.

### 7.3 High perpendicular exchange bias in VAN films

Perpendicular exchange bias (PEB) is appealing for applications in perpendicular magnetic tunneling junctions (MTJs). Owing to PMA in VAN systems, PEB is also expected. Another advantage of VAN compared to traditional FM/AFM bilayers or SLs, where a *H*_E_ ∼ 1/*t*_F_ law (where *H*_E_ and *t*_F_ are the EB field and film thickness) is obeyed, is that exchange bias (EB) scales with *t*_F_, because the coupled vertical interfacial area scales with *t*_F_.

The first trial PEB system was a (BiFeO_3_)_0.9_–(Fe_3_O_4_)_0.1_ composite film (Fe_3_O_4_ nanoparticles in a BiFeO_3_ film). A PEB field of *H*_E_ ∼ 0.04 kOe was obtained at room temperature.^112*b*^ Later, larger PEB values were obtained in LaFeO_3_–La_0.7_Sr_0.3_MnO_3_ and BiFeO_3_–La_0.7_Sr_0.3_MnO_3_ VAN systems with EB fields up to ∼1 kOe,^112*c*,*d*^ although this was achieved at a relatively low temperatures (<10 K). Most recently, a large PEB of ∼0.91 kOe at room temperature was obtained in the NiO–NiFe_2_O_4_ VAN system (Fig. 7d).^22^ With the benefits of both high *T*_C_ and high *T*_N_ of the FM and AF materials as well as *T*_C_ > *T*_N_, the blocking temperature (*T*_B_) in this system reached above 400 K.

## 8. Multiferroics and the magnetoelectric effect in VAN films

The key aims for non-volatile memory devices are large ME coupling coefficient at room temperature, and self-biased characteristics (electric field control of magnetism and no applied magnetic field). While, there are many reviews on both SL and VAN for creating artificial multiferroics, *i.e.* use of coupled systems rather than an intrinsic single phase material,^135^ there has been insufficient information discussing the problems of achieving ‘practical’ systems using two different materials forms and how to overcome these. We cover these points below.

A strong advantage of VAN artificial multiferroics over SL are minimal clamping from the underlying substrate. Clamping prevents effective vertical strain and strain coupling of a ferroelectric with a magnetostrictive material to produce a change in magnetisation with applied voltage to give the converse magnetoelectric effect (CME), or change in polarisation with change in magnetisation to give the direct magnetoelectric effect (DME).

**Fig. 8:**
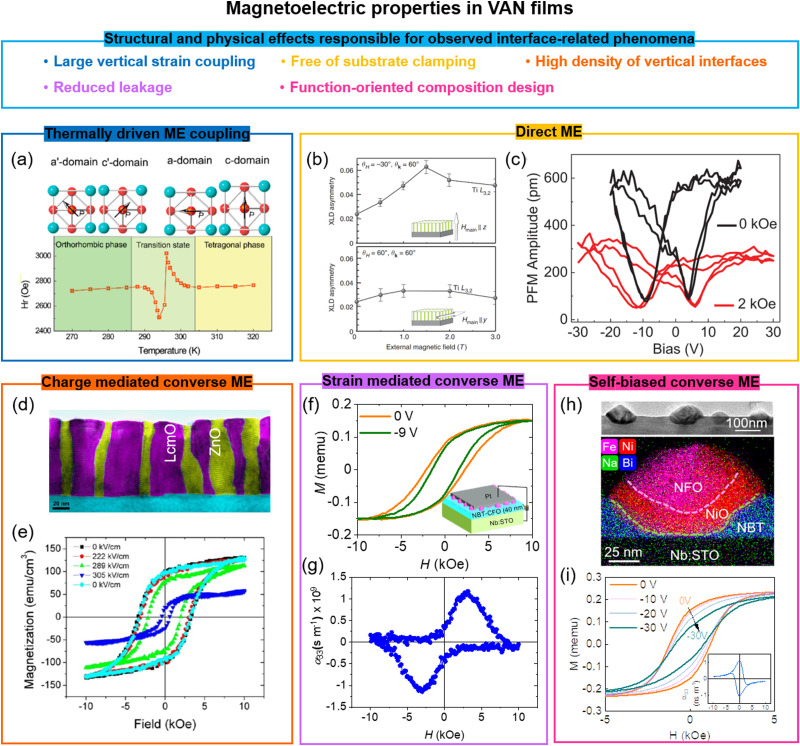
Enhanced magnetoelectric properties achieved using VAN structures. (a) Thermally-driven ME effect in Y_3_Fe_5_O_12_–BaTiO_3_ VAN films detected by the FMR. Reproduced with permission.^137^ Copyright 2017 American Chemical Society. (b) XLD asymmetry study of the lattice distortion induced by magnetic field in a BaTiO_3_–CoFe_2_O_4_ VAN film, the upper plot showing magnetic field applied OOP and the lower plot showing the magnetic field applied IP. Reproduced with permission.^138*a*^ Copyright 2013 Springer Nature. (c) The effect of the application of IP magnetic field on the amplitude–voltage butterfly loop (DME effect) in BaTiO_3_–CoFe_2_O_4_ VAN films. Reproduced with permission.^138*b*^ Copyright 2019 Wiley-VCH. (d and e) Charge mediated ME to give the CME effect in La_2_CoMnO_6_–ZnO VAN film. Reproduced with permission.^142^ Copyright 2013 American Chemical Society. (d) Cross-sectional (false-colour) TEM image of the La_2_CoMnO_6_–ZnO VAN film. (e) Electric field dependence of the magnetic hysteresis loops of La_2_CoMnO_6_–ZnO VAN film where the magnetic field is applied IP. (f) *In situ* electric field control of magnetization (CME effect) in Na_0.5_Bi_0.5_TiO_3_–CoFe_2_O_4_ VAN film where the magnetic field is applied OOP. Reproduced with permission.^143^ Copyright 2018 American Chemical Society. The inset shows a schematic of the application of an *in situ* electric field. (g) The magnetoelectric coefficient of the Na_0.5_Bi_0.5_TiO_3_–CoFe_2_O_4_ VAN film calculated from the magnetic hysteresis loops given in (f). (h) Cross-sectional TEM and EDX image of a 50 nm-thick Na_0.5_Bi_0.5_TiO_3_–NiO–NiFe_2_O_4_ film. Reproduced with permission.^40^ Copyright 2021 Springer Nature. (i) *In situ* electric field control of magnetization (CME effect) in a 200 nm-thick Na_0.5_Bi_0.5_TiO_3_–NiO–NiFe_2_O_4_ VAN film where the magnetic field is applied OOP. The inset shows the magnetoelectric coefficient, calculated from the magnetic hysteresis loops. Reproduced with permission.^40^ Copyright 2021 Springer Nature.

BaTiO_3_–CoFe_2_O_4_ was the first VAN film explored for achieving the ME effect.^130^ However, owing to poor electrical insulation of most ferromagnetic materials in multiferroic nanocomposites, only thermally-driven ME coupling was demonstrated. On the other hand, it has been shown in many different VAN systems *e.g.* BaTiO_3_–CoFe_2_O_4_,^130^ Ni_0.5_Zn_0.5_Fe_2_O_4_–BaTiO_3_^136^ and Y_3_Fe_5_O_12_–BaTiO_3_^137^ (Fig. 8a). However, thermal ME provides only indirect evidence of ME coupling, as the strain is not induced by an electric field.

In terms of DME, the BaTiO_3_–CoFe_2_O_4_ system is a key exemplar system.^138^ Here, magnetic field induced strain has been observed using different methods, X-ray linear dichroism (XLD) (Fig. 8b),^138*a*^ second-harmonic generation,^138*b*^ and by measuring the modified PFM amplitude upon applying a magnetic field along both IP and OOP directions (Fig. 8c).^138*b*,139^ However, possibly owing to leakage problems, a direct electric readout of DME has not been reported.

The first direct measurement of the CME effect was shown in BiFeO_3_–CoFe_2_O_4_ VAN.^140^ Here, magnetic hysteresis loops were measured to detect the change of magnetization upon applying *ex situ* electric field pulses. However, this system suffered leakage as revealed by conductive atomic force microscopy (c-AFM) and cross-sectional scanning tunnelling microscopy (XSTM).^15*a*,141^

In La_2_CoMnO_6_–ZnO VAN films, *in situ* measurements showed the existence of the CME effect as well as tunability of magnetization and coercivity (Fig. 8d and e).^142^ It was determined that voltage-controlled charge trapping-detrapping in the ZnO at the high-density vertical interfaces led to a modified transition metal ion valence of Mn or Co in the La_2_CoMnO_6_ and hence to a change in the magnetic state of the ferrimagnetic La_2_CoMnO_6_. However, the low *T*_C_ of La_2_CoMnO_6_ (<210 K) in the thin films meant that the CME was observed only at a relatively low temperature (120 K).

The first direct measurement of a large CME effect at room temperature was observed in a VAN system of Na_0.5_Bi_0.5_TiO_3_–CoFe_2_O_4_. By using the wide bandgap (3.3 eV), high *T*_C_ (603 K) Na_0.5_Bi_0.5_TiO_3_ ferroelectric instead of the commonly studied, lower *T*_C_ and lower bandgap BaTiO_3_ and BiFeO_3_ ferroelectrics, a large-scale *in situ* electric field control of the magnetic anisotropy was obtained at room temperature (Fig. 8f).^143^ The relatively low leakage was linked to (a) the lower leakage ferroelectric, (b) reduction of VAN vertical interface leakage which can arise when mixed valence transition metal cations are present in the system, and most importantly (c) the formation of a rectifying interface between the film and the substrate, enabling current blockage. An CME coefficient of around 1.25 × 10^−9^ S m^−1^, at a bias field of ∼0.3 T, was achieved (Fig. 8g).

Taking the above result one step further, a function-oriented composition design approach was used to reduce leakage even more. Hence, an antiferromagnetic (AFM) phase was incorporated into a similar VAN composition as discussed above,^40^ making a triple composite system. Here, a triple VAN 3-1 structure of FE-AFM-FM Na_0.5_Bi_0.5_TiO_3_–NiO–NiFe_2_O_4_ was created (Fig. 8h). We note that AFM materials have so far played an important role in traditional spintronic devices as pinning layers,^144^ and as emerging AFM spintronic devices, offering ultrafast (∼THz) dynamics, high stability to external fields, and absence of stray fields.^145^ However, they have largely been overlooked in multiferroic VANs. In the triple VAN system, the high *T*_C_ (603 K) of Na_0.5_Bi_0.5_TiO_3_ was again exploited, as above. But in addition, the soft magnetism of NiFe_2_O_4_, the high Néel temperature (525 K) of NiO, and the large exchange coupling between the FM and AFM material was also capitalised upon.

A large, self-biased (zero applied magnetic field) CME effect at room temperature with a ME coefficient of up to 1.38 × 10^−9^ s m^−1^ was achieved (Fig. 8i). The self-biased CME effect is ascribed to 3 critical features introduced by the addition of NiO: the leakage is strongly suppressed by the introduction of p-type NiO; the antiferromagnetic spins are rotated by the strain due to the large magnetostrictive effect of NiO, as indicated by the electric controllable EB effect; and the exchange coupling between the AFM NiO spins and the FM NiFe_2_O_4_ spins enhance the overall ME effect. This one-shot growth film, which does not rely on precise control of composition since if one phase has a slightly different ratio than another it will not be of detriment to the coupling effect, holds much promise for wide ranging applications relating to non-volatile memory and magnetic sensors.^146^ The next steps for this work are to demonstrate growth on TiN/Si.

## C. Summary, perspectives and conclusions

## 9. Summary of ferroic phenomena in VAN films

VAN films formed from complex oxides provide an excellent platform for achieving enhanced ferroic properties in thin films by a one-shot process, *i.e.* the creating of self-assembled composite films. So far, most of the VAN systems have focused on strain coupling effects across the vertical interfaces, which give rise to 3D strain tuning of the bulk of the film, thus tuning the film properties up to micron thickness levels. The anisotropic nature of the nanopillars in VAN films can enable magnetic anisotropy effects to be tuned. VANs are different to the SL films where most emphasis has been on emergent interface properties and much thinner films. There has been much less emphasis on emergent interface effects in VAN films and there is a strong opportunity to explore more in this direction. The challenges for VAN are that there are limitations on what materials can be combined together, that the interfaces are non-ideal 2D, and that there may be more than one interface type in a given system. Overall, VAN films have strong promise for enhancing properties of ferroic films which are required to be relatively thick (*e.g.* actuator/energy harvesting), anisotropic (*e.g.* for perpendicular magnetic recording), or unclamped (*e.g.* multiferroic).

## 10. Future perspectives

Throughout the paper, we highlighted the different scientific discoveries and ferroic functionalities arising in oxide superlattice (SL) and self-assembled vertically aligned nanocomposite (VAN) structures. Using exemplars, we also discussed how and why the two systems offer different and often complementary effects. Below, we highlight some underexplored/emerging research directions for ferroic properties in both oxide superlattices and VAN systems.

### 10.1 Future perspective for superlattice films

1. Although many SL examples discussed have great potential for the discovery of new physical phenomena, one must always be aware of the many subtleties in dealing with these intrinsically nanoscale systems. As summarized in Fig. 1 and Table 1, in terms of tuning the functionalities of superlattice films, defects (cationic intermixing, point defect segregation, structural inhomogeneities, oxygen vacancies and so on) all play important, often poorly understood and interlinked roles. More work employing high intensity spectroscopic tools should be employed to understand the nature of the interface structures. Also, in order to minimise the role of interfacial strain which often produces a defective interface, focus on systems with minimal interfacial mismatch should be explored. This can be done by using new single crystal substrate compositions. There has been some excellent work in this direction in recent years.^147^

Also, to minimise strain and defect formation, the choice of more closely lattice matched layers in the SL films should be explored, where possible. To minimise cation non-stoichiometry in films, growth under lower oxidation conditions should be studied.^148^ To minimise defects, films could be grown higher temperatures (*i.e.* at above 1000 °C rather than the conventional 700–800 °C).^149^ However, considering a key driver is to make oxide SLs CMOS compatible, other strategies to minimise defects at ∼500 °C and below must also be considered, *e.g.* atomic layer growth methods where epitaxial perovskite films have been grown at <300 °C,^150^ as well as novel ways to enhance growth kinetics and also carefully control composition.^14^

2. Most ferroic SLs have been grown mainly on perovskite substrates with (001)-orientation. Epitaxial integration of ferroic superlattices with different crystal orientation other than (001) should be explored. This field has grown rapidly in the past decade but there are many more materials combinations to be explored. Non-(001) orientations give different surface/interface terminations, neutral charge states and 2D-like structures (*e.g.* (111) orientations giving buckled honeycombs). Also, growth on a range of buffered semiconductor substrates beyond the well-studied Si, *e.g.* on Ge, Si–Ge, GaAs, GaN, and for flexible electronics, flexible substrates, *e.g.* mica^151^ or analogous crystals needs to be explored.

3. Room-temperature ferroelectricity and ferromagnetism have been widely achieved in superlattice films. However, room-temperature multiferroic superlattices with strong and robust magnetoelectric coupling have still not been realized. Since lift-off procedures for oxide films are now quite well established,^86,91,152^ this allows clamping effects to be overcome and so more work could be directed here to achieve robust, crack-free, non-wrinkled and well adhered films. Also, the vast majority of work on superlattices has focused on *A*/*B* superlattices. However, there is a largely unexplored area of multicomponent superlattices with complex periodicities. These could offer much further potential for achieving interesting ferroic behaviour. An example was shown for tricolor superlattices in Fig. 4, where three constituent materials forming an *ABC* periodicity. With the *ABC* periodicity, inversion symmetry is broken, enabling interesting novel magnetoelectric phenomena to be achieved.

4. There remains much to be understood about emergent phenomena, such as polar skyrmions, vortices, negative capacitance, and so on. Real-space topological textures such as polar skyrmions and vortices have been observed, but we are still in the early stages of the exploration of these phenomena. For example, the dynamics of such skyrmions structures and vortices must be explored in detail. In addition, these topologies demonstrate chirality and if this can be manipulated by an external field (such as an electric field), then this would provide a powerful way to use the chirality as an externally controllable order parameter.

5. The interfaces between 3d TMOs and 5d TMOs provide a new fruitful research area. Again, current research is still in the early stages and limited to a few systems, so more systematic investigations are highly desired to fully unravel the unique role of 5d TMOs which in turn could provide more candidates to search for novel magnetic textures and topological phenomena. Moreover, owing to the inherent mixture of spin and orbital degrees of freedom in 5d TMOs, these superlattices may open a new pathway to achieve robust electric field control of magnetism at room temperature and above.

### 10.2 Future perspective for VAN films

1. Just as for SL films, VAN films are subject to a range of interface and bulk point and line defects, such as mutual doping of components.^153^ To further optimise the perfection of these structures, similar application of spectroscopic tools, and the implementation of carefully controlled growth processes combined with a range of theoretical predictions of microstructure evolution, should take place.

2. As highlighted in Table 1, the wide ranging emergent interface phenomena, topological, skyrmion and polar vortices observed in SL films have not been observed in VAN films. Considering the very high epitaxial quality of VAN films, the ultraclean self-assembled interfaces, and the ability to control dimensionalities down to a few nm *via* controlling growth conditions, there are unique opportunities to discover and control these novel effects in VAN.

3. Combining VAN and SLs to form 3D film architectures has not yet been widely studied.^154^ However, these structures could offer the chance to combine the complementary advantages of both methods and should be explored further.

4. Pulsed laser deposition is the main method to make VAN films with good quality. However, the method is area-limited. However, promising growth of a limited number of VAN systems by sputtering^155^ and chemical methods^156^ show the possibility for growth by methods of industrial interest. More work should be focused in these areas.

5. As already noted, compared to SL films, VAN films can exhibit more bulk-like effects, *i.e.* interface-driven effects throughout the whole film, not just right at the interfaces. Their simplicity of fabrication is also an important aspect. However, typically they have a random column distribution. For some applications, such as data storage, a highly ordered arrangement is desired. Templating by a variety of ways can produce the desired order,^2*b*,155*a*,157^ recently spontaneous order has been shown.^2*b*^ More work is needed to determine which methods are most practical for applications.

6. Just as for SLs, mentioned above, most VAN film work has been undertaken on single crystal substrates although progress has been made with STO/TiN-buffered Si^158^ and with mica,^159^ and lifted-off films onto Si.^92^ More work is required on these’other substrates’ as well as new ways to grow directly on Si, *e.g.* using remote epitaxy.

7. As shown in Section 6, high crystalline quality mesoporous films derived from VAN films give enhanced ferroelectric effects and they also exploit high area surface effects for photocatalysis. There are many new opportunities to exploit the large surface area effects or to create new composites (not just oxide–oxide but also oxide–metal and oxide–organic systems) by chemical coating of mesoporous films made by selective chemical etching of VAN films. With this broader range of compositions, new coupling effects in wide ranging functional systems can be realised.

## 11. Conclusions

Over more than three decades, fascinating physical phenomena have been achieved in heteroepitaxial interfaces in complex oxide superlattices (SLs), and over the last two decades in vertically aligned nanocomposites (VANs). Numerous novels or enhanced ferroic phenomena have been achieved which have been highlighted in this review. The subtlety of the different natures of the interfaces in these two materials forms, SL or VAN, enables highly complementary phenomena to be achieved. Notably, SL films with 2D interfaces enable basic emergent physical phenomena to be discovered and explored, for example the creation of ferroelectricity,^11,12,15,16^ ferromagnetism,^41,42,45^ and multiferroism,^63^ emergent polar skyrmions and vortices,^25,27^*etc.* On the other hand, VAN films offer greater simplicity of growth, cleaner self-assembled interfaces, are largely unclamped by the substrate, and can be grown to micron thickness. On the other hand, they have more complex geometric vertical interfaces, and a much narrower range of materials can be grown. VAN are more suited to the engineering of ferroic properties, *e.g.* to increase the ferroelectric *T*_C_,^21,54^ to increase the magnetization^34,115,119^ and to enhance the magnetoelectric coupling.^130,143,160^ For both material forms, there are many new avenues to be explored for property discoveries and the creation of practical engineering devices, with several examples highlighted in this review, *e.g.* topological textures, negative capacitance, integration with silicon and other semiconductor substrates, flexibilization, *etc.*

## Conflicts of interest

There are no conflicts of interest to declare.

## Supplementary Material
